# A two-sample Bayesian *t*-test for microarray data

**DOI:** 10.1186/1471-2105-7-126

**Published:** 2006-03-10

**Authors:** Richard J Fox, Matthew W Dimmic

**Affiliations:** 1 Codexis, Inc., Redwood City, CA 94063, USA; 2 Department of Biological Statistics and Computational Biology, Cornell University, Ithaca, NY 14853, USA; 3 Divergence, Inc., St. Louis, MO 63141, USA

## Abstract

**Background:**

Determining whether a gene is differentially expressed in two different samples remains an important statistical problem. Prior work in this area has featured the use of *t*-tests with pooled estimates of the sample variance based on similarly expressed genes. These methods do not display consistent behavior across the entire range of pooling and can be biased when the prior hyperparameters are specified heuristically.

**Results:**

A two-sample Bayesian *t*-test is proposed for use in determining whether a gene is differentially expressed in two different samples. The test method is an extension of earlier work that made use of point estimates for the variance. The method proposed here explicitly calculates in analytic form the marginal distribution for the difference in the mean expression of two samples, obviating the need for point estimates of the variance without recourse to posterior simulation. The prior distribution involves a single hyperparameter that can be calculated in a statistically rigorous manner, making clear the connection between the prior degrees of freedom and prior variance.

**Conclusion:**

The test is easy to understand and implement and application to both real and simulated data shows that the method has equal or greater power compared to the previous method and demonstrates consistent Type I error rates. The test is generally applicable outside the microarray field to any situation where prior information about the variance is available and is not limited to cases where estimates of the variance are based on many similar observations.

## Background

Determining whether a gene is differentially expressed under different conditions is an important statistical problem [1-3]. Consider, for example, a common microarray experiment: for a particular gene *Y*, one has an unordered set of measurements of log-expression levels y1c⋯yn1c
 MathType@MTEF@5@5@+=feaafiart1ev1aaatCvAUfKttLearuWrP9MDH5MBPbIqV92AaeXatLxBI9gBaebbnrfifHhDYfgasaacH8akY=wiFfYdH8Gipec8Eeeu0xXdbba9frFj0=OqFfea0dXdd9vqai=hGuQ8kuc9pgc9s8qqaq=dirpe0xb9q8qiLsFr0=vr0=vr0dc8meaabaqaciaacaGaaeqabaqabeGadaaakeaacqWG5bqEdaqhaaWcbaGaeGymaedabaGaem4yamgaaOGaeS47IWKaemyEaK3aa0baaSqaaiabd6gaUnaaBaaameaacqaIXaqmaeqaaaWcbaGaem4yamgaaaaa@380F@ in a control situation and y1t⋯yn2t
 MathType@MTEF@5@5@+=feaafiart1ev1aaatCvAUfKttLearuWrP9MDH5MBPbIqV92AaeXatLxBI9gBaebbnrfifHhDYfgasaacH8akY=wiFfYdH8Gipec8Eeeu0xXdbba9frFj0=OqFfea0dXdd9vqai=hGuQ8kuc9pgc9s8qqaq=dirpe0xb9q8qiLsFr0=vr0=vr0dc8meaabaqaciaacaGaaeqabaqabeGadaaakeaacqWG5bqEdaqhaaWcbaGaeGymaedabaGaemiDaqhaaOGaeS47IWKaemyEaK3aa0baaSqaaiabd6gaUnaaBaaameaacqaIYaGmaeqaaaWcbaGaemiDaqhaaaaa@3855@ in a treatment situation (or any non-control situation) (notation similar to [4]). The question of interest is whether any expression difference Δy¯=∑j=1n2yjt/n2−∑i=1n1yic/n1=y¯2−y¯1
 MathType@MTEF@5@5@+=feaafiart1ev1aaatCvAUfKttLearuWrP9MDH5MBPbIqV92AaeXatLxBI9gBaebbnrfifHhDYfgasaacH8akY=wiFfYdH8Gipec8Eeeu0xXdbba9frFj0=OqFfea0dXdd9vqai=hGuQ8kuc9pgc9s8qqaq=dirpe0xb9q8qiLsFr0=vr0=vr0dc8meaabaqaciaacaGaaeqabaqabeGadaaakeaacqqHuoarcuWG5bqEgaqeaiabg2da9maaqahabaGaemyEaK3aa0baaSqaaiabdQgaQbqaaiabdsha0baakiabc+caViabd6gaUnaaBaaaleaacqaIYaGmaeqaaaqaaiabdQgaQjabg2da9iabigdaXaqaaiabd6gaUnaaBaaameaacqaIYaGmaeqaaaqdcqGHris5aOGaeyOeI0YaaabCaeaacqWG5bqEdaqhaaWcbaGaemyAaKgabaGaem4yamgaaOGaei4la8IaemOBa42aaSbaaSqaaiabigdaXaqabaaabaGaemyAaKMaeyypa0JaeGymaedabaGaemOBa42aaSbaaWqaaiabigdaXaqabaaaniabggHiLdGccqGH9aqpcuWG5bqEgaqeamaaBaaaleaacqaIYaGmaeqaaOGaeyOeI0IafmyEaKNbaebadaWgaaWcbaGaeGymaedabeaaaaa@58CD@ is a significant difference, or if it would be expected under the null hypothesis of no actual difference in expression.

Though multifactorial experimental designs are becoming increasingly popular [5-7], there continue to be experimentalists interested in analyzing two-sample designs. There are many approaches to determining whether genes are differentially expressed in such designs and there are a number of excellent reviews of the various methods [2,3]. Many of the approaches include the use of a *t*-test [8-14], which is a common frequentist statistical approach to comparing a difference in sample means. Some have pointed out that the normality assumption used in *t*-tests may not always hold and other statistical techniques may be warranted [15,16]. Even for those measurements where normality holds the *t*-test is difficult to apply because the number of replicates *n*_1 _and *n*_2 _are often quite small (typically, *n*_1 _= *n*_2 _= 1 to 3), leading to uncertainty in the sample variance and relatively low power. An early approach to dealing with this problem was based on the addition of a constant value to the estimate of the standard deviation [16,17]. While the approach does tend to regularize the variance estimates, it is *ad hoc *and is not expected to exhibit statistically consistent behavior (unknown or incorrect Type I error rate under the null).

More rigorous Bayesian methods have been developed that incorporate prior information about the variance but require Markov chain sampling of the posterior density or numerical integration of the cumulative distribution function [18-21]. A very popular work by Baldi and Long [4] avoids such calculations while using a statistically justified regularization technique via construction of a probabilistic Bayesian framework that applies a prior probability to parameters of interest such as the expression means and variances. An analytically tractable solution can then be obtained by taking a point estimate for the variance. The chief advantages of this Bayesian framework are twofold: it allows the use of a regularized *t*-test to determine whether a difference in expression is significant, and it provides a natural method for incorporating information from putatively related measurements. It is this basic framework that we seek to extend in the work presented here.

As noted by Baldi and Long [4], the use of a point estimate for the variance is a compromise between a rigorous Bayesian approach and tractable calculation. As demonstrated below, this compromise can lead to bias in the specificity of the test and improper behavior when there are few prior degrees of freedom. Our hope here is to correct this and other issues (both theoretical and practical) towards the goal of improving the performance and understanding of microarray and general laboratory data analysis. To address these issues, we extend the basic framework put forth earlier, taking the model to a fully Bayesian approach by explicitly calculating the marginal posterior distribution for the difference in mean expression levels. This analytically tractable solution does not require simulation from the posterior distribution and obviates the need to use point estimates of the prior variance while overcoming the undesirable correlation between specificity and prior degrees of freedom. Moreover, a clear and statistically rigorous connection between the prior variance and the prior degrees of freedom is established, reducing the number of hyperparameters from two to one while yielding consistent Type I error rates in the process. It should be stressed that achieving consistent Type I error rates is not just a conceptual luxury. Indeed proper determination of important quantities such as the false discovery rate are of great economical significance and rely on having correct Type I error rates [22].

## Method

### Bayesian probability model

The original model proposed by Baldi and Long [4] is briefly reviewed here. The likelihood of the observed data, **y**, given the true expression level, μ, and variance, σ^2^, for a single gene is assumed to follow a normal distribution:

p(y|μ,σ2)∝∏i=1nN(yi;μ,σ2)     (1)
 MathType@MTEF@5@5@+=feaafiart1ev1aaatCvAUfKttLearuWrP9MDH5MBPbIqV92AaeXatLxBI9gBaebbnrfifHhDYfgasaacH8akY=wiFfYdH8Gipec8Eeeu0xXdbba9frFj0=OqFfea0dXdd9vqai=hGuQ8kuc9pgc9s8qqaq=dirpe0xb9q8qiLsFr0=vr0=vr0dc8meaabaqaciaacaGaaeqabaqabeGadaaakeaacqWGWbaCcqGGOaakieqacqWF5bqEcqGG8baFcqaH8oqBcqGGSaalcqaHdpWCdaahaaWcbeqaaiabikdaYaaakiabcMcaPiabg2Hi1oaarahabaGaemOta4KaeiikaGIaemyEaK3aaSbaaSqaaiabdMgaPbqabaGccqGG7aWocqaH8oqBcqGGSaalcqaHdpWCdaahaaWcbeqaaiabikdaYaaakiabcMcaPaWcbaGaemyAaKMaeyypa0JaeGymaedabaGaemOBa4ganiabg+GivdGccaWLjaGaaCzcamaabmaabaGaeGymaedacaGLOaGaayzkaaaaaa@50DB@

where *n *is the number of measurements in one sample. For the priors on μ and σ^2 ^the authors attempt to capture the *a priori *dependence between μ and σ^2 ^by factoring the joint prior as *p*(μ,σ^2^) = *p*(μ|σ^2^)*p*(σ^2^) and taking each factor as

*p*(μ|σ^2^) = *N*(μ;μ_0_,σ^2^/λ_0_)     (2)

and

*p*(σ^2^) = *I*(σ^2^;ν_0_,σ02
 MathType@MTEF@5@5@+=feaafiart1ev1aaatCvAUfKttLearuWrP9MDH5MBPbIqV92AaeXatLxBI9gBaebbnrfifHhDYfgasaacH8akY=wiFfYdH8Gipec8Eeeu0xXdbba9frFj0=OqFfea0dXdd9vqai=hGuQ8kuc9pgc9s8qqaq=dirpe0xb9q8qiLsFr0=vr0=vr0dc8meaabaqaciaacaGaaeqabaqabeGadaaakeaacqaHdpWCdaqhaaWcbaGaeGimaadabaGaeGOmaidaaaaa@307C@)     (3)

where the prior probability of μ follows a normal distribution and σ^2 ^follows a scaled inverse gamma distribution. The hyperparameters μ_0 _and λ_0 _represent the prior beliefs regarding the value of μ and the confidence associated with that belief, while the hyperparameters σ02
 MathType@MTEF@5@5@+=feaafiart1ev1aaatCvAUfKttLearuWrP9MDH5MBPbIqV92AaeXatLxBI9gBaebbnrfifHhDYfgasaacH8akY=wiFfYdH8Gipec8Eeeu0xXdbba9frFj0=OqFfea0dXdd9vqai=hGuQ8kuc9pgc9s8qqaq=dirpe0xb9q8qiLsFr0=vr0=vr0dc8meaabaqaciaacaGaaeqabaqabeGadaaakeaacqaHdpWCdaqhaaWcbaGaeGimaadabaGaeGOmaidaaaaa@307C@ and ν_0 _represent the prior beliefs regarding the value of σ^2 ^and the degrees of freedom or confidence associated with that belief. The authors subsequently let λ_0 _→ 0 later in their derivation, leading to a diffuse, noninformative prior on μ:

lim⁡λ0→0p(μ|σ2)∝1σ     (4)
 MathType@MTEF@5@5@+=feaafiart1ev1aaatCvAUfKttLearuWrP9MDH5MBPbIqV92AaeXatLxBI9gBaebbnrfifHhDYfgasaacH8akY=wiFfYdH8Gipec8Eeeu0xXdbba9frFj0=OqFfea0dXdd9vqai=hGuQ8kuc9pgc9s8qqaq=dirpe0xb9q8qiLsFr0=vr0=vr0dc8meaabaqaciaacaGaaeqabaqabeGadaaakeaadaWfqaqaaiGbcYgaSjabcMgaPjabc2gaTbWcbaGaeq4UdW2aaSbaaWqaaiabicdaWaqabaWccqGHsgIRcqaIWaamaeqaaOGaemiCaaNaeiikaGIaeqiVd0MaeiiFaWNaeq4Wdm3aaWbaaSqabeaacqaIYaGmaaGccqGGPaqkcqGHDisTdaWcaaqaaiabigdaXaqaaiabeo8aZbaacaWLjaGaaCzcamaabmaabaGaeGinaqdacaGLOaGaayzkaaaaaa@4805@

The remaining 1/σ factor that comes from a diffuse normal results in a prior of the form *p*(μ,σ^2^) ∝ σ^-1^*I*(σ^2^;ν_0_,σ02
 MathType@MTEF@5@5@+=feaafiart1ev1aaatCvAUfKttLearuWrP9MDH5MBPbIqV92AaeXatLxBI9gBaebbnrfifHhDYfgasaacH8akY=wiFfYdH8Gipec8Eeeu0xXdbba9frFj0=OqFfea0dXdd9vqai=hGuQ8kuc9pgc9s8qqaq=dirpe0xb9q8qiLsFr0=vr0=vr0dc8meaabaqaciaacaGaaeqabaqabeGadaaakeaacqaHdpWCdaqhaaWcbaGaeGimaadabaGaeGOmaidaaaaa@307C@). Using Bayes' rule the authors obtain the following posterior distribution:

*p*(μ,σ^2^|**y**,α) ∝ *N*(μ;μ_*n*_,σ^2^/λ_*n*_)*I*(σ^2^;ν_*n*_,σn2
 MathType@MTEF@5@5@+=feaafiart1ev1aaatCvAUfKttLearuWrP9MDH5MBPbIqV92AaeXatLxBI9gBaebbnrfifHhDYfgasaacH8akY=wiFfYdH8Gipec8Eeeu0xXdbba9frFj0=OqFfea0dXdd9vqai=hGuQ8kuc9pgc9s8qqaq=dirpe0xb9q8qiLsFr0=vr0=vr0dc8meaabaqaciaacaGaaeqabaqabeGadaaakeaacqaHdpWCdaqhaaWcbaGaemOBa4gabaGaeGOmaidaaaaa@30F3@)     (5)

where α is a vector of hyperparameters (μ_0_,λ_0_,ν_0_,σ02
 MathType@MTEF@5@5@+=feaafiart1ev1aaatCvAUfKttLearuWrP9MDH5MBPbIqV92AaeXatLxBI9gBaebbnrfifHhDYfgasaacH8akY=wiFfYdH8Gipec8Eeeu0xXdbba9frFj0=OqFfea0dXdd9vqai=hGuQ8kuc9pgc9s8qqaq=dirpe0xb9q8qiLsFr0=vr0=vr0dc8meaabaqaciaacaGaaeqabaqabeGadaaakeaacqaHdpWCdaqhaaWcbaGaeGimaadabaGaeGOmaidaaaaa@307C@) for the prior and

μn=λ0λ0+nμ0+nλ0+ny¯     (6)
 MathType@MTEF@5@5@+=feaafiart1ev1aaatCvAUfKttLearuWrP9MDH5MBPbIqV92AaeXatLxBI9gBaebbnrfifHhDYfgasaacH8akY=wiFfYdH8Gipec8Eeeu0xXdbba9frFj0=OqFfea0dXdd9vqai=hGuQ8kuc9pgc9s8qqaq=dirpe0xb9q8qiLsFr0=vr0=vr0dc8meaabaqaciaacaGaaeqabaqabeGadaaakeaacqaH8oqBdaWgaaWcbaGaemOBa4gabeaakiabg2da9maalaaabaGaeq4UdW2aaSbaaSqaaiabicdaWaqabaaakeaacqaH7oaBdaWgaaWcbaGaeGimaadabeaakiabgUcaRiabd6gaUbaacqaH8oqBdaWgaaWcbaGaeGimaadabeaakiabgUcaRmaalaaabaGaemOBa4gabaGaeq4UdW2aaSbaaSqaaiabicdaWaqabaGccqGHRaWkcqWGUbGBaaGafmyEaKNbaebacaWLjaGaaCzcamaabmaabaGaeGOnaydacaGLOaGaayzkaaaaaa@48B4@

λ_*n *_= λ_0 _+ *n *    (7)

ν_*n *_= ν_0 _+ *n *    (8)

νnσn2=ν0σ02+(n−1)s2+λ0nλ0+n(y¯−μ0)2     (9)
 MathType@MTEF@5@5@+=feaafiart1ev1aaatCvAUfKttLearuWrP9MDH5MBPbIqV92AaeXatLxBI9gBaebbnrfifHhDYfgasaacH8akY=wiFfYdH8Gipec8Eeeu0xXdbba9frFj0=OqFfea0dXdd9vqai=hGuQ8kuc9pgc9s8qqaq=dirpe0xb9q8qiLsFr0=vr0=vr0dc8meaabaqaciaacaGaaeqabaqabeGadaaakeaacqaH9oGBdaWgaaWcbaGaemOBa4gabeaakiabeo8aZnaaDaaaleaacqWGUbGBaeaacqaIYaGmaaGccqGH9aqpcqaH9oGBdaWgaaWcbaGaeGimaadabeaakiabeo8aZnaaDaaaleaacqaIWaamaeaacqaIYaGmaaGccqGHRaWkcqGGOaakcqWGUbGBcqGHsislcqaIXaqmcqGGPaqkcqWGZbWCdaahaaWcbeqaaiabikdaYaaakiabgUcaRmaalaaabaGaeq4UdW2aaSbaaSqaaiabicdaWaqabaGccqWGUbGBaeaacqaH7oaBdaWgaaWcbaGaeGimaadabeaakiabgUcaRiabd6gaUbaacqGGOaakcuWG5bqEgaqeaiabgkHiTiabeY7aTnaaBaaaleaacqaIWaamaeqaaOGaeiykaKYaaWbaaSqabeaacqaIYaGmaaGccaWLjaGaaCzcamaabmaabaGaeGyoaKdacaGLOaGaayzkaaaaaa@5ACA@

The sufficient statistics are y¯=∑i=1nyi/n
 MathType@MTEF@5@5@+=feaafiart1ev1aaatCvAUfeBSjuyZL2yd9gzLbvyNv2CaerbwvMCKfMBHbqedmvETj2BSbqee0evGueE0jxyaibaieYdOi=BH8vipeYdI8qiW7rqqrFfpeea0xe9Lq=Jc9vqaqpepm0xbbG8FasPYRqj0=yi0lXdbba9pGe9qqFf0dXdHuk9fr=xfr=xfrpiWZqaaeaabiGaaiaacaqabeaabeqacmaaaOqaaiqadMhagaqeaiabg2da9maaqahabaGaamyEamaaBaaaleaacaWGPbaabeaakiaac+cacaWGUbaaleaacaWGPbGaeyypa0JaaGymaaqaaiaad6gaa0GaeyyeIuoaaaa@3B8A@ and s2=∑i=1n(yi−y¯)2/(n−1)
 MathType@MTEF@5@5@+=feaafiart1ev1aaatCvAUfKttLearuWrP9MDH5MBPbIqV92AaeXatLxBI9gBaebbnrfifHhDYfgasaacH8akY=wiFfYdH8Gipec8Eeeu0xXdbba9frFj0=OqFfea0dXdd9vqai=hGuQ8kuc9pgc9s8qqaq=dirpe0xb9q8qiLsFr0=vr0=vr0dc8meaabaqaciaacaGaaeqabaqabeGadaaakeaacqWGZbWCdaahaaWcbeqaaiabikdaYaaakiabg2da9maaqahabaGaeiikaGIaemyEaK3aaSbaaSqaaiabdMgaPbqabaGccqGHsislcuWG5bqEgaqeaiabcMcaPmaaCaaaleqabaGaeGOmaidaaOGaei4la8IaeiikaGIaemOBa4MaeyOeI0IaeGymaeJaeiykaKcaleaacqWGPbqAcqGH9aqpcqaIXaqmaeaacqWGUbGBa0GaeyyeIuoaaaa@4583@. Finally, the authors calculate the posterior mean estimate or point estimate (PE) of the variance for one sample as

σ2=νnνn−2σn2     (10)
 MathType@MTEF@5@5@+=feaafiart1ev1aaatCvAUfKttLearuWrP9MDH5MBPbIqV92AaeXatLxBI9gBaebbnrfifHhDYfgasaacH8akY=wiFfYdH8Gipec8Eeeu0xXdbba9frFj0=OqFfea0dXdd9vqai=hGuQ8kuc9pgc9s8qqaq=dirpe0xb9q8qiLsFr0=vr0=vr0dc8meaabaqaciaacaGaaeqabaqabeGadaaakeaacqaHdpWCdaahaaWcbeqaaiabikdaYaaakiabg2da9maalaaabaGaeqyVd42aaSbaaSqaaiabd6gaUbqabaaakeaacqaH9oGBdaWgaaWcbaGaemOBa4gabeaakiabgkHiTiabikdaYaaacqaHdpWCdaqhaaWcbaGaemOBa4gabaGaeGOmaidaaOGaaCzcaiaaxMaadaqadaqaaiabigdaXiabicdaWaGaayjkaiaawMcaaaaa@422F@

This estimate is then used in the hypothesis testing procedures implemented in their Cyber-T software. Though this model is developed using a Bayesian framework to make a point estimate of the variance, the hypothesis testing is carried out using a classical frequentist *t*-test.

In the standard *t*-test, the variance is still defined when there are no prior degrees of freedom, but inspection of Eq. (10) shows that when the number of prior degrees of freedom ν_0 _< 2 - *n*, the variance is undefined. This limiting behavior stems from the use of a point estimate for the variance. The total number of prior degrees of freedom used in the Cyber-T software is actually 2ν_0 _as it counts ν_0 _prior degrees of freedom for each sample in the two-sample test. However, for consistency and clarity we hereafter refer to the total number of prior degrees of freedom as ν_0_. Therefore the total number of degrees of freedom used in the Cyber-T software for the two-sample hypothesis test then appears to be ν_*t-test *_= ν_0 _+ *n*_1 _+ *n*_2 _- 4, where *n*_1 _is the number of experimental replicates of the gene in the control regime and *n*_2 _is the number of replicates of the gene in the treatment regime. This equation for the total degrees of freedom also demonstrates undesired limiting behavior when ν_0 _= 0, since positive degrees of freedom exist only when *n*_1 _+ *n*_2 _> 4. This result contradicts what we would expect to see from a classic two-sample *t*-test with no prior estimate of the variance, i.e. a classical two-sample *t*-test has degrees of freedom *n*_1 _+ *n*_2 _- 2 and positive degrees of freedom exist when *n*_1 _+ *n*_2 _> 2.

### Full two-sample model

#### Posterior distribution

Instead of using a point estimate for the variance to conduct a frequentist *t*-test, we can recast the problem to directly infer the posterior distribution for a two-sample case:

*p*(μ,Δμ,σ^2^|**y**) ∝ *p*(μ,Δμ,σ^2^)*p*(**y**|μ,Δμ,σ^2^)     (11)

where μ is the mean expression level of the gene in the control regime, μ +Δμ the mean of the gene in the alternate experimental regime, and σ^2 ^the variance associated with the measurements. In contrast to the previous, point estimate model, in this formulation there is no explicit prior dependence between σ^2 ^and μ. While this is admittedly a simplification, this independence is not only a practical requirement for the full Bayesian integration, we also believe it is justified on several other grounds. First, while it has been widely observed that genes with different expression levels often have different variances [1], it is not clear how the prior used by Baldi and Long [4] to derive the point estimate of variance could capture the dependencies observed with actual microarray data. Their figures show that the logarithm of the mean expression level tends to decrease with increasing variance, yet the prior given by Eq. (2) asserts that the prior probability of the mean should be more diffuse – not necessarily smaller – with increasing variance. The observed trend between mean and variance cannot be captured by such a prior. Second, their method does include an *implicit *dependence between mean and variance by using other gene expression levels to determine the priors; indeed, this is what lends the method its power. Because the Bayesian formulation proposed here determines the prior hyperparameters in a similar fashion, it retains this implicit dependence. One may also consider a number of variance stabilizing transforms that may be applied in order to achieve constant variance across the expression spectrum [23,24]. Finally, as shown below, simulations reveal that the method still retains power even when the true variance is allowed to vary over a substantial range.

Returning to the posterior distribution, we assume that the samples from each experimental regime follow a normal distribution, each with equal variances and (possibly) different means.

*y*_*i *_~ *N*(μ,σ^2^)     (12)

*y*_*j *_~ *N*(μ + Δμ,σ^2^)     (13)

This leads to the following posterior distributionp

p(μ,Δμ,σ2|y)∝p(μ,Δμ,σ2)∏i=1n112πσexp⁡(−12σ2(yi−μ)2)∏j=1n212πσexp⁡(−12σ2((yj−(μ+Δμ))2)     (14)
 MathType@MTEF@5@5@+=feaafiart1ev1aaatCvAUfKttLearuWrP9MDH5MBPbIqV92AaeXatLxBI9gBaebbnrfifHhDYfgasaacH8akY=wiFfYdH8Gipec8Eeeu0xXdbba9frFj0=OqFfea0dXdd9vqai=hGuQ8kuc9pgc9s8qqaq=dirpe0xb9q8qiLsFr0=vr0=vr0dc8meaabaqaciaacaGaaeqabaqabeGadaaakeaafaqabeGabaaabaGaemiCaaNaeiikaGIaeqiVd0MaeiilaWIaeuiLdqKaeqiVd0MaeiilaWIaeq4Wdm3aaWbaaSqabeaacqaIYaGmaaGccqGG8baFtCvAUfeBSjuyZL2yd9gzLbvyNv2CaeHbwvMCKfMBHbaceeGaa8xEaiabcMcaPiabg2Hi1cqaaiabdchaWjabcIcaOiabeY7aTjabcYcaSiabfs5aejabeY7aTjabcYcaSiabeo8aZnaaCaaaleqabaGaeGOmaidaaOGaeiykaKYaaebCaeaadaWcaaqaaiabigdaXaqaamaakaaabaGaeGOmaiJaeqiWdaNaeq4WdmhaleqaaaaaaeaacqWGPbqAcqGH9aqpcqaIXaqmaeaacqWGUbGBdaWgaaadbaGaeGymaedabeaaa0Gaey4dIunakiGbcwgaLjabcIha4jabcchaWnaabmaabaGaeyOeI0YaaSaaaeaacqaIXaqmaeaacqaIYaGmcqaHdpWCdaahaaWcbeqaaiabikdaYaaaaaGccqGGOaakcqWG5bqEdaWgaaWcbaGaemyAaKgabeaakiabgkHiTiabeY7aTjabcMcaPmaaCaaaleqabaGaeGOmaidaaaGccaGLOaGaayzkaaWaaebCaeaadaWcaaqaaiabigdaXaqaamaakaaabaGaeGOmaiJaeqiWdaNaeq4WdmhaleqaaaaaaeaacqWGQbGAcqGH9aqpcqaIXaqmaeaacqWGUbGBdaWgaaadbaGaeGOmaidabeaaa0Gaey4dIunakiGbcwgaLjabcIha4jabcchaWnaabmaabaGaeyOeI0YaaSaaaeaacqaIXaqmaeaacqaIYaGmcqaHdpWCdaahaaWcbeqaaiabikdaYaaaaaGcdaqadaqaaiabcIcaOiabdMha5naaBaaaleaacqWGQbGAaeqaaOGaeyOeI0IaeiikaGIaeqiVd0Maey4kaSIaeuiLdqKaeqiVd0MaeiykaKcacaGLOaGaayzkaaWaaWbaaSqabeaacqaIYaGmaaaakiaawIcacaGLPaaaaaGaaCzcaiaaxMaadaqadaqaaiabigdaXiabisda0aGaayjkaiaawMcaaaaa@A2CD@

where *n*_1 _and *n*_2 _are the number of measurements in each sample. We elect not to make use of the prior on μ used in the Baldi work, Eq. (2), or its limiting form, Eq. (4). Instead, we assume a completely flat prior on both μ and Δμ while retaining the prior for σ^2 ^given by Eq. (3). Similar to the earlier work, the observed dependence between μ and σ^2 ^is established by calculating σ02
 MathType@MTEF@5@5@+=feaafiart1ev1aaatCvAUfKttLearuWrP9MDH5MBPbIqV92AaeXatLxBI9gBaebbnrfifHhDYfgasaacH8akY=wiFfYdH8Gipec8Eeeu0xXdbba9frFj0=OqFfea0dXdd9vqai=hGuQ8kuc9pgc9s8qqaq=dirpe0xb9q8qiLsFr0=vr0=vr0dc8meaabaqaciaacaGaaeqabaqabeGadaaakeaacqaHdpWCdaqhaaWcbaGaeGimaadabaGaeGOmaidaaaaa@307C@ based on the variance of similarly expressed genes. However, in this formulation, instead of taking the average standard deviation of similarly expressed genes we choose to estimate the prior variance by totaling the sum-squared differences for each similar gene and dividing by the total prior degrees of freedom. This is a more statistically rigorous way of incorporating prior information and leads to more a more consistent test as will be discussed below.

#### Marginal posterior for Δμ

With the above definitions and assumptions in place, it can be shown (see Appendix) that the marginal posterior distribution follows a *t *distribution:

Δμ−Δy¯σn1n1+1n2|y∼tνn     (15)
 MathType@MTEF@5@5@+=feaafiart1ev1aaatCvAUfKttLearuWrP9MDH5MBPbIqV92AaeXatLxBI9gBaebbnrfifHhDYfgasaacH8akY=wiFfYdH8Gipec8Eeeu0xXdbba9frFj0=OqFfea0dXdd9vqai=hGuQ8kuc9pgc9s8qqaq=dirpe0xb9q8qiLsFr0=vr0=vr0dc8meaabaqaciaacaGaaeqabaqabeGadaaakeaadaabcaqaamaalaaabaGaeuiLdqKaeqiVd0MaeyOeI0IaeuiLdqKafmyEaKNbaebaaeaacqaHdpWCdaWgaaWcbaGaemOBa4gabeaakmaakaaabaWaaSaaaeaacqaIXaqmaeaacqWGUbGBdaWgaaWcbaGaeGymaedabeaaaaGccqGHRaWkdaWcaaqaaiabigdaXaqaaiabd6gaUnaaBaaaleaacqaIYaGmaeqaaaaaaeqaaaaaaOGaayjcSdGaemyEaKNaeSipIOJaemiDaq3aaSbaaSqaaiabe27aUnaaBaaameaacqWGUbGBaeqaaaWcbeaakiaaxMaacaWLjaWaaeWaaeaacqaIXaqmcqaI1aqnaiaawIcacaGLPaaaaaa@4D11@

where

ν_*n *_= *n*_1 _+ *n*_2 _+ ν_0 _- 2     (16)

νnσn2=ν0σ02+(n1−1)s12+(n2−1)s22     (17)
 MathType@MTEF@5@5@+=feaafiart1ev1aaatCvAUfKttLearuWrP9MDH5MBPbIqV92AaeXatLxBI9gBaebbnrfifHhDYfgasaacH8akY=wiFfYdH8Gipec8Eeeu0xXdbba9frFj0=OqFfea0dXdd9vqai=hGuQ8kuc9pgc9s8qqaq=dirpe0xb9q8qiLsFr0=vr0=vr0dc8meaabaqaciaacaGaaeqabaqabeGadaaakeaacqaH9oGBdaWgaaWcbaGaemOBa4gabeaakiabeo8aZnaaDaaaleaacqWGUbGBaeaacqaIYaGmaaGccqGH9aqpcqaH9oGBdaWgaaWcbaGaeGimaadabeaakiabeo8aZnaaDaaaleaacqaIWaamaeaacqaIYaGmaaGccqGHRaWkcqGGOaakcqWGUbGBdaWgaaWcbaGaeGymaedabeaakiabgkHiTiabigdaXiabcMcaPiabdohaZnaaDaaaleaacqaIXaqmaeaacqaIYaGmaaGccqGHRaWkcqGGOaakcqWGUbGBdaWgaaWcbaGaeGOmaidabeaakiabgkHiTiabigdaXiabcMcaPiabdohaZnaaDaaaleaacqaIYaGmaeaacqaIYaGmaaGccaWLjaGaaCzcamaabmaabaGaeGymaeJaeG4naCdacaGLOaGaayzkaaaaaa@55D1@

The sufficient statistics are Δy¯=∑j=1n2yj/n2−∑i=1n1yi/n1=y¯2−y¯1
 MathType@MTEF@5@5@+=feaafiart1ev1aaatCvAUfKttLearuWrP9MDH5MBPbIqV92AaeXatLxBI9gBaebbnrfifHhDYfgasaacH8akY=wiFfYdH8Gipec8Eeeu0xXdbba9frFj0=OqFfea0dXdd9vqai=hGuQ8kuc9pgc9s8qqaq=dirpe0xb9q8qiLsFr0=vr0=vr0dc8meaabaqaciaacaGaaeqabaqabeGadaaakeaacqqHuoarcuWG5bqEgaqeaiabg2da9maaqahabaGaemyEaK3aaSbaaSqaaiabdQgaQbqabaGccqGGVaWlcqWGUbGBdaWgaaWcbaGaeGOmaidabeaaaeaacqWGQbGAcqGH9aqpcqaIXaqmaeaacqWGUbGBdaWgaaadbaGaeGOmaidabeaaa0GaeyyeIuoakiabgkHiTmaaqahabaGaemyEaK3aaSbaaSqaaiabdMgaPbqabaGccqGGVaWlcqWGUbGBdaWgaaWcbaGaeGymaedabeaaaeaacqWGPbqAcqGH9aqpcqaIXaqmaeaacqWGUbGBdaWgaaadbaGaeGymaedabeaaa0GaeyyeIuoakiabg2da9iqbdMha5zaaraWaaSbaaSqaaiabikdaYaqabaGccqGHsislcuWG5bqEgaqeamaaBaaaleaacqaIXaqmaeqaaaaa@560B@, and the sum squared differences (*n*_1 _- 1)s12
 MathType@MTEF@5@5@+=feaafiart1ev1aaatCvAUfKttLearuWrP9MDH5MBPbIqV92AaeXatLxBI9gBaebbnrfifHhDYfgasaacH8akY=wiFfYdH8Gipec8Eeeu0xXdbba9frFj0=OqFfea0dXdd9vqai=hGuQ8kuc9pgc9s8qqaq=dirpe0xb9q8qiLsFr0=vr0=vr0dc8meaabaqaciaacaGaaeqabaqabeGadaaakeaacqWGZbWCdaqhaaWcbaGaeGymaedabaGaeGOmaidaaaaa@302A@ and (*n*_2 _- 1)s22
 MathType@MTEF@5@5@+=feaafiart1ev1aaatCvAUfKttLearuWrP9MDH5MBPbIqV92AaeXatLxBI9gBaebbnrfifHhDYfgasaacH8akY=wiFfYdH8Gipec8Eeeu0xXdbba9frFj0=OqFfea0dXdd9vqai=hGuQ8kuc9pgc9s8qqaq=dirpe0xb9q8qiLsFr0=vr0=vr0dc8meaabaqaciaacaGaaeqabaqabeGadaaakeaacqWGZbWCdaqhaaWcbaGaeGOmaidabaGaeGOmaidaaaaa@302C@, where s12=∑i=1n1(yi−y¯1)2/(n1−1)
 MathType@MTEF@5@5@+=feaafiart1ev1aaatCvAUfKttLearuWrP9MDH5MBPbIqV92AaeXatLxBI9gBaebbnrfifHhDYfgasaacH8akY=wiFfYdH8Gipec8Eeeu0xXdbba9frFj0=OqFfea0dXdd9vqai=hGuQ8kuc9pgc9s8qqaq=dirpe0xb9q8qiLsFr0=vr0=vr0dc8meaabaqaciaacaGaaeqabaqabeGadaaakeaacqWGZbWCdaqhaaWcbaGaeGymaedabaGaeGOmaidaaOGaeyypa0ZaaabCaeaacqGGOaakcqWG5bqEdaWgaaWcbaGaemyAaKgabeaakiabgkHiTiqbdMha5zaaraWaaSbaaSqaaiabigdaXaqabaGccqGGPaqkdaahaaWcbeqaaiabikdaYaaaaeaacqWGPbqAcqGH9aqpcqaIXaqmaeaacqWGUbGBdaWgaaadbaGaeGymaedabeaaa0GaeyyeIuoakiabc+caViabcIcaOiabd6gaUnaaBaaaleaacqaIXaqmaeqaaOGaeyOeI0IaeGymaeJaeiykaKcaaa@49D1@ and s22=∑j=1n2(yj−y¯2)2/(n2−1)
 MathType@MTEF@5@5@+=feaafiart1ev1aaatCvAUfKttLearuWrP9MDH5MBPbIqV92AaeXatLxBI9gBaebbnrfifHhDYfgasaacH8akY=wiFfYdH8Gipec8Eeeu0xXdbba9frFj0=OqFfea0dXdd9vqai=hGuQ8kuc9pgc9s8qqaq=dirpe0xb9q8qiLsFr0=vr0=vr0dc8meaabaqaciaacaGaaeqabaqabeGadaaakeaacqWGZbWCdaqhaaWcbaGaeGOmaidabaGaeGOmaidaaOGaeyypa0ZaaabCaeaacqGGOaakcqWG5bqEdaWgaaWcbaGaemOAaOgabeaakiabgkHiTiqbdMha5zaaraWaaSbaaSqaaiabikdaYaqabaGccqGGPaqkdaahaaWcbeqaaiabikdaYaaaaeaacqWGQbGAcqGH9aqpcqaIXaqmaeaacqWGUbGBdaWgaaadbaGaeGOmaidabeaaa0GaeyyeIuoakiabc+caViabcIcaOiabd6gaUnaaBaaaleaacqaIYaGmaeqaaOGaeyOeI0IaeGymaeJaeiykaKcaaa@49DD@. This distribution has the feature that the standard *t*-test is obtained even when there are no prior degrees of freedom, ν_0_ = 0, in which case the observed variance for each gene dominates the posterior density. A hypothesis test is performed by asserting a null hypothesis that the true difference in expression levels is zero, i.e. Δ*μ *= 0 (though other values for the true difference, Δ*μ*, can be used when attempting to identify genes that are differentially expressed by some threshold degree). When the posterior probability of no differential expression approaches zero, Pr(Δμ = 0|**y**) → 0, the null is rejected. It is worth noting that while the posterior probability distribution for no differential expression, Pr(Δμ = 0|**y**), follows the same frequentist distribution for the data, Pr(**y**|Δμ = 0) [25], the resulting probabilities have different interpretations. In the Bayesian approach the posterior probability is interpreted as the probability that there is no difference in expression levels. In the frequentist approach the probability is interpreted as the probability of observing such a difference from the null distribution of no differential expression given chance alone. When Δ*μ *= 0 the Bayesian and frequentist methods give the same numerical results and one can ignore the interpretational differences when discussing such things as the false positive rate under the null. However, power analysis calculations (Δ*μ *≠ 0) are best conducted under a frequentist framework using a non-central *t*-distribution [25].

#### Estimating the prior variance

For *m *similarly expressed genes each having *n *replicates, the prior degrees of freedom for the variance can be calculated as

ν_0 _= *m*(*n *- 1)     (18)

The prior variance is then calculated as the total sum-square differences for each sample of similarly expressed genes divided by the prior degrees of freedom, namely

σ02=∑k=1m∑i=1n(yk,i−y¯k)2ν0     (19)
 MathType@MTEF@5@5@+=feaafiart1ev1aaatCvAUfKttLearuWrP9MDH5MBPbIqV92AaeXatLxBI9gBaebbnrfifHhDYfgasaacH8akY=wiFfYdH8Gipec8Eeeu0xXdbba9frFj0=OqFfea0dXdd9vqai=hGuQ8kuc9pgc9s8qqaq=dirpe0xb9q8qiLsFr0=vr0=vr0dc8meaabaqaciaacaGaaeqabaqabeGadaaakeaacqaHdpWCdaqhaaWcbaGaeGimaadabaGaeGOmaidaaOGaeyypa0ZaaSaaaeaadaaeWbqaamaaqahabaGaeiikaGIaemyEaK3aaSbaaSqaaiabdUgaRjabcYcaSiabdMgaPbqabaGccqGHsislcuWG5bqEgaqeamaaBaaaleaacqWGRbWAaeqaaOGaeiykaKYaaWbaaSqabeaacqaIYaGmaaaabaGaemyAaKMaeyypa0JaeGymaedabaGaemOBa4ganiabggHiLdaaleaacqWGRbWAcqGH9aqpcqaIXaqmaeaacqWGTbqBa0GaeyyeIuoaaOqaaiabe27aUnaaBaaaleaacqaIWaamaeqaaaaakiaaxMaacaWLjaWaaeWaaeaacqaIXaqmcqaI5aqoaiaawIcacaGLPaaaaaa@5357@

where y¯k
 MathType@MTEF@5@5@+=feaafiart1ev1aaatCvAUfKttLearuWrP9MDH5MBPbIqV92AaeXatLxBI9gBaebbnrfifHhDYfgasaacH8akY=wiFfYdH8Gipec8Eeeu0xXdbba9frFj0=OqFfea0dXdd9vqai=hGuQ8kuc9pgc9s8qqaq=dirpe0xb9q8qiLsFr0=vr0=vr0dc8meaabaqaciaacaGaaeqabaqabeGadaaakeaacuWG5bqEgaqeamaaBaaaleaacqWGRbWAaeqaaaaa@2FCA@ is the mean response of gene *k *and *y*_*k*,*i *_is the response *i *of gene *k*. In general the prior degrees of freedom and variance should be determined using these equations and not chosen separately from one another. While it is tempting to think of the prior variance as separate from the precision or credibility associated with that estimate (as represented by ν_0_), a consistent hypothesis test requires that they be considered together based on the actual prior data collected.

## Results

To assess the performance of the new test, a series of simulations were done to measure the false positive rate and statistical power under various conditions. The simulations consisted of the following steps:

1) Draw random samples from two normal distributions of size *n*_1 _and *n*_2 _distributed as *N*(μ_1_,σtest2
 MathType@MTEF@5@5@+=feaafiart1ev1aaatCvAUfKttLearuWrP9MDH5MBPbIqV92AaeXatLxBI9gBaebbnrfifHhDYfgasaacH8akY=wiFfYdH8Gipec8Eeeu0xXdbba9frFj0=OqFfea0dXdd9vqai=hGuQ8kuc9pgc9s8qqaq=dirpe0xb9q8qiLsFr0=vr0=vr0dc8meaabaqaciaacaGaaeqabaqabeGadaaakeaacqaHdpWCdaqhaaWcbaGaemiDaqNaemyzauMaem4CamNaemiDaqhabaGaeGOmaidaaaaa@3532@) and *N*(μ_2_,σtest2
 MathType@MTEF@5@5@+=feaafiart1ev1aaatCvAUfKttLearuWrP9MDH5MBPbIqV92AaeXatLxBI9gBaebbnrfifHhDYfgasaacH8akY=wiFfYdH8Gipec8Eeeu0xXdbba9frFj0=OqFfea0dXdd9vqai=hGuQ8kuc9pgc9s8qqaq=dirpe0xb9q8qiLsFr0=vr0=vr0dc8meaabaqaciaacaGaaeqabaqabeGadaaakeaacqaHdpWCdaqhaaWcbaGaemiDaqNaemyzauMaem4CamNaemiDaqhabaGaeGOmaidaaaaa@3532@) respectively. This step represents the selection of two different experimental regimes for a gene of interest in order to test for differential expression. For power testing, the standardized effect (μ_1 _- μ_2_)/σ_*test *_= 2 is used to generate the data by setting μ_1 _= 2, μ_2 _= 0, and σ_*test *_= 1. For false positive rate testing μ_1 _= 0.

2) Draw an estimate of the prior variance from the following Chi-square distribution: σ02~σtest2χν2/ν0
 MathType@MTEF@5@5@+=feaafiart1ev1aaatCvAUfKttLearuWrP9MDH5MBPbIqV92AaeXatLxBI9gBaebbnrfifHhDYfgasaacH8akY=wiFfYdH8Gipec8Eeeu0xXdbba9frFj0=OqFfea0dXdd9vqai=hGuQ8kuc9pgc9s8qqaq=dirpe0xb9q8qiLsFr0=vr0=vr0dc8meaabaqaciaacaGaaeqabaqabeGadaaakeaacqaHdpWCdaqhaaWcbaGaeGimaadabaGaeGOmaidaaOGaeiOFa4Naeq4Wdm3aa0baaSqaaiabdsha0jabdwgaLjabdohaZjabdsha0bqaaiabikdaYaaakiabeE8aJnaaDaaaleaacqaH9oGBaeaacqaIYaGmaaGccqGGVaWlcqaH9oGBdaWgaaWcbaGaeGimaadabeaaaaa@42EA@. This step simulates the task of estimating the prior variance from similarly expressed genes. It is mathematically equivalent to calculating the sum-squared differences of similarly expressed genes and dividing by their prior degrees of freedom, but it is computationally more efficient.

3) Perform hypothesis tests using a) the method used by Baldi and Long [4] based on the mean posterior point estimate for the variance, PE(σ^2^) or just PE, and b) the new test proposed here based on the marginal posterior for the difference in expression level, MP(Δμ) or just MP. The hypothesis tests using each method are done at the α = 0.05 level using a two-sided, two-sample *t*-test. Test *p*-values below this level are counted as significant. To make valid comparisons, both methods used the same number of prior degrees of freedom.

4) Steps 1 to 3 above are repeated 10,000 times.

Results of the power simulations for a number of sample sizes and prior degrees of freedom are shown in Table 1. Because the PE test cannot be used when ν_0 _+ *n*_1 _+ *n*_2 _≤ 4, the test has no power and no false positive rate when *n*_1 _= *n*_2 _= 2 and ν_0 _= 0. Conversely, the MP test results in a classical *t*-test in this case and has a power of 0.2169 and a false positive rate of 0.0509, which is near the expected value given the α = 0.05 level of the test. Holding *n*_1 _= *n*_2 _= 2 but increasing the number of prior degrees of freedom to ν_0 _= 2 (equal to 2 other similarly expressed genes used to determine the prior variance) the PE test can now be used but has a very low power of 0.0450, compared to the MP power of 0.3343. Overall, when the estimate of the variance is balanced between the prior and observed variances (i.e. the prior number of degrees of freedom is not large compared to the number of replicates in the test samples) the MP test is significantly more powerful. The difference in power between the two methods is less pronounced when ν_0 _= 16 (the default value used in the Cyber-T software when *n*_1 _= *n*_2 _= 2). However, one should note that such a relatively high number for the prior degrees of freedom represents a strong degree of belief in the prior estimate of the variance relative to the variance in the genes of interest.

**Table 1:** Comparison of false positive rate and statistical power for fixed α. For a given significance level (α) the observed false positive rate (FP) and power were determined by simulation using either a point estimate for the variance (PE) or the marginal posterior distribution for Δμ (MP). Values are given for different sample sizes (*n*_1 _= *n*_2 _= *n*) and prior degrees of freedom (ν_0_).

*n*_1 _= *n*_2 _= *n*	ν_0_	α_PE_	α_MP_	FP_PE_	FP_MP_	Power_PE_	Power_MP_
2	0	0.05	0.05	-	0.0509	-	0.2169
2	2	0.05	0.05	0.0044	0.0490	0.0450	0.3343
2	4	0.05	0.05	0.0146	0.0535	0.1814	0.3919
2	6	0.05	0.05	0.0211	0.0518	0.2747	0.4182
2	16	0.05	0.05	0.0385	0.0498	0.4186	0.4709
3	0	0.05	0.05	0.0030	0.0500	0.0709	0.4664
3	2	0.05	0.05	0.0167	0.0486	0.2833	0.5404
3	4	0.05	0.05	0.0261	0.0556	0.4153	0.5834
3	6	0.05	0.05	0.0280	0.0500	0.4838	0.6053
3	16	0.05	0.05	0.0389	0.0501	0.5951	0.6441

These simulations reveal that the observed false positive rate for the PE test is substantially out of line with the nominal value of 0.05 for small sample replicates (*n*_1 _= *n*_2 _≤ 3) and few prior degrees of freedom (ν_0 _≤ 16). Conversely, the MP test demonstrates consistent behavior for the observed false positive rate. However, it is possible to obtain the desired false positive rate for the PE method by iterating through values of α. Table 2 shows that, by adjusting the critical α value for the PE method until the observed false positive rate matches that of the MP method, both tests have similar power. The disadvantage is that α no longer represents the expected Type I error rate, and simulation or calibration is required whenever a new test is performed. Conversely, with the MP test, the critical value α represents an unbiased estimate of the Type I error rate.

**Table 2:** Comparison of α and statistical power for iterated false positive rate. The values are the same as those given in Table 1 except the significance level (α) was iterated for the PE method until its power matched that of the MP method where α was held constant.

*n*_1 _= *n*_2 _= *n*	ν_0_	α_PE_	α_MP_	FP_PE_	FP_MP_	Power_PE_	Power_MP_
2	0	-	0.05	-	0.0505	-	0.2146
2	2	0.190	0.05	0.0514	0.0489	0.3374	0.3336
2	4	0.120	0.05	0.0483	0.0522	0.3783	0.3840
2	6	0.095	0.05	0.0496	0.0514	0.4302	0.4267
2	16	0.067	0.05	0.0491	0.0508	0.4794	0.4738
3	0	0.190	0.05	0.0507	0.0513	0.4576	0.4599
3	2	0.115	0.05	0.0531	0.0462	0.5324	0.5308
3	4	0.095	0.05	0.0475	0.0486	0.5611	0.5641
3	6	0.082	0.05	0.0536	0.0500	0.5994	0.6053
3	16	0.066	0.05	0.0511	0.0472	0.6531	0.6469

It is interesting to look for a simple correction to the degrees of freedom in order to make the two methods match each other. Unfortunately, there does not appear to be any simple correction available. The degrees of freedom used in the *t*-test itself would be easy to modify – though *ad hoc*, one could change the last term in the expression for the degrees of freedom used in the PE test (ν_*t-test *_= ν_0 _+ *n*_1 _+ *n*_2 _- 4) from -4 to -2 by simply adding two to ν_0_. Unfortunately, the point estimate of the variance given by Eq. (10) is more problematic as it appears to give an increased estimate of the variance compared to the one used in the MP test, resulting in a decrease in both the *t*-statistic and the statistical power.

In the Cyber-T software [4] the prior variance is estimated by averaging the standard deviation over 100 similarly expressed genes. This has two problems associated with it. First, the measured standard deviation is a biased estimate and tends to be smaller than the true value for small sample sizes like *n *= 2 or 3, the average of these measured standard deviations will also be biased downward and in general E(σ2)≠E(σ2)
 MathType@MTEF@5@5@+=feaafiart1ev1aaatCvAUfKttLearuWrP9MDH5MBPbIqV92AaeXatLxBI9gBaebbnrfifHhDYfgasaacH8akY=wiFfYdH8Gipec8Eeeu0xXdbba9frFj0=OqFfea0dXdd9vqai=hGuQ8kuc9pgc9s8qqaq=dirpe0xb9q8qiLsFr0=vr0=vr0dc8meaabaqaciaacaGaaeqabaqabeGadaaakeaacqWGfbqrcqGGOaakdaGcaaqaaiabeo8aZnaaCaaaleqabaGaeGOmaidaaaqabaGccqGGPaqkcqGHGjsUdaGcaaqaaiabdweafnaabmaabaGaeq4Wdm3aaWbaaSqabeaacqaIYaGmaaaakiaawIcacaGLPaaaaSqabaaaaa@39D7@, where *E*(·) represents the expected value of a random variable. The correct way to estimate the variance based on prior observations is to calculate the total sum square differences for *all *the prior samples and divide by the total degrees of freedom, as shown in Eq. (19). Second, if similarly expressed genes for each of the two samples are used to estimate the prior variance and they have the same number of replicates, *n*, as the samples themselves, then estimating the standard deviation from *m *= 100 similarly expressed genes is tantamount to asserting ν_0 _= 100·(*n *- 1) prior degrees of freedom. Setting ν_0 _to less than the actual number of prior degrees of freedom used to calculate the prior variance results in a lower actual false positive rate than the rejection level α. For consistency if we fix the number of prior degrees of freedom that are used in the test, then we must choose a corresponding number of similarly expressed genes that together have the same number of prior degrees of freedom. This dependence between ν_0 _and *m *is more consistent with the formulation of the prior itself and leads to consistent Type I error rates. When we calculate the prior variance in the statistically rigorous manner (by taking the total sum square differences of prior observations and dividing by the total prior degrees of freedom) the PE method becomes overly conservative, generating false positives with a rate of 0.0044 at *n*_1 _= *n*_2 _= 2, α = 0.05, and ν_0 _= 2, this appears to be the result of using a point estimate for the prior variance that is larger than the expected value given similar genes, see Eq. (10).

As mentioned previously, to perform the full Bayesian integration required by the MP test, the assumption of a constant variance is required. To test the effect of violating this assumption, simulations were performed as described above, but in step 2 the variances for each sample were drawn from a uniform distribution in the range [0.05,1]. The same uniform distribution was used to generate prior variances by calculating the total sum-squared differences of *m *= ν_0_/(*n *- 1) different draws (corresponding to *m *different genes) for the variance and dividing by the prior degrees of freedom, ν_0_. The results for both the PE and MP methods are shown in Table 3, where the observed false positive rate for the MP method is used to calibrate the α value for the PE test so as to achieve nearly the same false positive rate for each method. Though both methods are seen to have nearly the same power for a given false positive rate, the PE method again shows large deviations between α and the observed false positive rate. However, the MP method shows more consistency between the observed and theoretical rates and is fairly robust to violations of the constant variance assumption.

**Table 3:** Comparison of α and statistical power for iterated false positive rate and variable variance. The values are the same as those given in Table 1 and Table 2 except the variance for each sample was drawn from a uniform distribution between 0.05 and 1 during the simulation. The significance level (α) for the PE method was iterated so that the observed false positive rate matched that of the MP method where α was held constant.

*n*_1 _= *n*_2 _= *n*	ν_0_	α_PE_	α_MP_	FP_PE_	FP_MP_	Power_PE_	Power_MP_
2	0	-	0.05	-	0.0554	-	0.4038
2	2	0.190	0.05	0.0593	0.0592	0.5876	0.5756
2	6	0.090	0.05	0.0522	0.0521	0.6838	0.6859
2	16	0.067	0.05	0.0573	0.0571	0.7563	0.7566

To test whether these statistical improvements in the method translate to improvements in experimental data analysis, both methods were evaluated with the same microarray data [26] used by Baldi and Long [27]. The Arfin data matrix is composed of 8 columns by 1973 rows that contain gene expression levels used to study IHF regulated genes in *Escherichia coli*. Columns 1 to 4 correspond to 4 IHF^+ ^strains while columns 5 to 8 correspond to 4 IHF^- ^strains. The 1973 rows correspond to the measured gene expression levels for each strain. There are actually more rows in the original data set but to be consistent with the earlier work, we only chose rows that had measurable expression levels for all 8 runs. As was done in the Long paper the 4 control and 4 treatment replicates were partitioned into 12 subsets that differ by at least two replicates so that quasi-independent pairwise comparisons could be made (i.e. 12v56, 12v78, 13v57, 13v68, 14v58, 14v67, 23v58, 23v67, 24v57, 24v68, 34v56, and 34v78). For the experimental evaluation we adopted the same strategy for pooling variances used by Baldi and Long. We sorted all the genes within each sample by the mean expression level and computed the prior variance by considering a window of the *m*/2 most similarly expressed genes around each gene of interest. For the two samples this corresponds to a total of *m *similarly expressed genes and Eq. (18) gives the total prior degrees of freedom, ν_0 _= *m*.

To measure the consistency of a given testing method the number of common genes discovered between two quasi-independent subsets can be recorded. Genes that are discovered to be in common between subsets are more likely to be truly differentially expressed, thus higher a commonality percentage (genes discovered in common divided by the total number of genes discovered) is an indication that the testing method is more reliable. The number of common genes discovered can be recorded for the 66 possible ways of comparing the 12 subsets. Based on *p*-value rankings, the MP method achieved similar results (within sampling error) to those published using the Cyber-T software. The single hyperparameter (ν_0_) was optimized for the MP method and a 95% confidence interval of [62.4, 67.8] common genes were identified out of the top 120. This compares well with the published results using Cyber-T where optimizing two parameters independently (ν_0 _and *m*) achieved an average common gene count of 67. These results represent a substantial improvement over the 33 common genes identified using a zero parameter classical *t*-test.

Because the *p*-value rankings by themselves are not actually statistical tests, a more stringent approach to assessing the performance of each method was accomplished by identifying genes that are differentially expressed at some confidence level α. For the following tests we used a *p*-value of 0.01 for the null hypothesis cutoff (uncorrected for multiple tests). The results are presented in Table 4. In general, for a given number of prior degrees of freedom, the MP method consistently detects more genes as differentially expressed than does the PE method and more of these genes are commonly discovered when comparing them with quasi-independent subsets. In addition the fraction of common genes within those detected is generally higher for the MP method. The difference between the methods tends to decrease as the number of prior degrees of freedom is increased. The optimal performance (in terms of % commonality) is achieved at around ν_0 _= 400, which corresponds to basing the local variance on about 10% of the 1972 similarly expressed genes within each sample (there are 3944 similarly expressed genes when considering both samples). As ν_0 _increases past 400 the commonality percentage drops somewhat. Basing the variance on the combined estimated of all 3944 similar genes results in a difference-in-means test, and the drop in performance is similar to that seen by Long and Baldi when a simple difference in means was used to rank the expression differences [27].

**Table 4:** Experimental comparison of hypothesis testing methods. The microarray data consists of 1973 genes partitioned into 12 quasi independent *n*_1 _= 2 and *n*_2 _= 2 subsets using replicate experiments of 4 control and 4 treatment runs [26, 27]. The number of genes detected as differentially expressed using each method is listed for an α = 0.01 significance level (there was no correction for multiple testing) and different prior degrees of freedom (ν_0_). The average number of common genes for the 66 unique comparisons of the 12 subsets is also listed along with the % of commonality (common/detected) for each test.

ν_0_	Detected_PE_	Detected_MP_	Common_PE_	Common_MP_	% Common_PE_	% Common_MP_
0	0	46.2	0	6.4	0	13.8
2	3.3	72.5	0.2	21.0	6.3	29.0
4	29.7	88.0	8.7	30.9	29.2	35.1
8	61.5	95.2	23.4	37.9	38.1	39.8
16	83.3	103.9	34.0	42.6	40.1	40.1
400	107.0	108.1	51.2	51.5	47.9	47.6
1972	118.8	119.0	54.7	54.7	46.0	46.0
3944	109.1	109.2	48.6	48.7	44.6	44.6

It is important to stress that the above results for the PE method are not what one would expect using the implementation found in the Cyber-T software [4]. As mentioned before, this is because the existing software incorrectly calculates the pooled variance by averaging the standard deviation of similarly expressed genes. This results in a substantial error for example, when *n*_1 _= *n*_2 _= 2 and ν_0 _= 400, the Type I error rate is approximately 0.125 (2.5 times higher than that predicted by the nominal rate α = 0.05). The authors of Cyber-T recommend smaller values of ν_0 _where the overestimate of variance given by their point estimate, Eq. (10), is balanced by the underestimate of variance owing to incorrect pooling, thereby achieving (approximately) correct false positive rates by offsetting two competing biases.

## Discussion and conclusion

The new marginal posterior (MP) formulation proposed here is uniformly at least as powerful as the previous point estimate (PE) method which it is based on and more powerful when the number of similarly expressed genes (and therefore prior degrees of freedom ν_0_) is small. Moreover, unlike the existing regularization methods, the new formulation consistently maintains the proper false positive rates under the null hypothesis across the entire range of pooling. The MP formulation also makes clear the dependence between the prior degrees of freedom and prior variance and thus methods to estimate the single hyperparameter ν_0 _are expected to be more robust and interpretable.

As mentioned previously, the key to the power of these Bayesian methods is the use of similarly-expressed genes to estimate the expected variance. Naturally, the more similar the variance in expression of the other genes is to the gene of interest, the greater the statistical consistency of the method. In generating the simulated data we have attempted to remain agnostic on the definition of "similarly-expressed", using variances that are either constant or drawn from a uniform distribution. Another Bayesian method [28] utilizes a clustering technique based on a series of *t*-statistics to group genes into prior categories in order to pool their prior variances. Because the Bayesian posterior probabilities used by that method are not interpretable as frequentist Type I error rates, it is difficult to set the desired false positive rate using such a method without resorting to simulation and iteration. Other clustering strategies include those based on non-parametric regression to obtain local variance estimates [29] and mixture models that group together similar variances [30] using maximum likelihood. These clustering strategies assume the variance is known based on the large number of observations in each group. This allows normal distributions to be used in deriving test statistics. Unfortunately, when the number of similarly expressed genes is small the use of a normal distribution is not justified as it assumes there is no uncertainty in the variance. The advantage of the MP formulation is that the normal approximation for the test statistic is not required – it is statistically robust across the entire range of pooling or clustering strategies (assuming the strategies work properly to group together genes of similar variance). In our experimental application of the method we have adopted a pooling strategy based on grouping together genes with similar expression levels. Thus the MP formulation can be used in situations where two or more control replicates can be compared with only single observations under treatment conditions; this is not possible with clustering strategies that require at least two replicates in each sample [30].

Though the MP test was developed in a Bayesian framework, there is a strong analogy with frequentist statistics. One person's prior is another person's data; if we treat the prior estimates of variance as just additional data collection then our hypothesis test using the *t *distribution is the same one as that used in any introductory statistics course. In either situation, one considers the estimate of variance as the total sum-of-squared differences for all observations of the variance divided by the total degrees of freedom. By "all observations" we mean both the two samples involved in the hypothesis test plus some number of replicates from other genes we believe to be similarly expressed and are good representatives of the variance. This is just like estimating the method standard deviation and conducting a normal *t*-test with that estimate along with the additional degrees of freedom that come with it. All of the power analysis calculations can then be done without recourse to simulations; for example, built-in functions in the desktop statistical package R (using the non-central *t *distribution) gave the same results we present by simulation for the MP method. It should be stressed that this Bayesian hypothesis test is not limited in any way to the analysis of microarray data. It can be applied to any experimental situation where prior beliefs about the variance associated with small sample sizes are used to achieve higher statistical power while maintaining consistent Type I error rates. This is particularly important when only a few similar observations of the variance are available, for example when only a handful of genes are under study across a range of treatment conditions.

The method is implemented in R and is freely available for download [31] or by contacting the corresponding author.

## Authors' contributions

RJF conceived of and derived the statistical formulation, applied the method to various data sets and drafted the manuscript. MWD contributed major revisions to the manuscript and suggested ways of improving the study. Both authors contributed to the final version of the manuscript and approved it.

## Appendix

### Determining the marginal posterior distribution of Δμ

The posterior is the product of the prior and the likelihood:

*p*(μ,Δμ,σ^2^|*y*) ∝ *p*(μ,Δμ,σ^2^)*p*(*y*|μ,Δμ,σ^2^)     (20)

The likelihood consists of the product of two normal distributions:

*y*_*i *_~ *N*(μ,σ_2_)     (21)

*y*_*j *_~ *N*(μ + Δμ,σ^2^)     (22)

p(μ,Δμ,σ2|y)∝p(μ,Δμ,σ2)∏i=1n112πσexp⁡(−12σ2(yi−μ)2)∏j=1n212πσexp⁡(−12σ2(yj−(μ+Δμ))2)     (23)
 MathType@MTEF@5@5@+=feaafiart1ev1aaatCvAUfKttLearuWrP9MDH5MBPbIqV92AaeXatLxBI9gBaebbnrfifHhDYfgasaacH8akY=wiFfYdH8Gipec8Eeeu0xXdbba9frFj0=OqFfea0dXdd9vqai=hGuQ8kuc9pgc9s8qqaq=dirpe0xb9q8qiLsFr0=vr0=vr0dc8meaabaqaciaacaGaaeqabaqabeGadaaakeaafaqabeGabaaabaGaemiCaaNaeiikaGIaeqiVd0MaeiilaWIaeuiLdqKaeqiVd0MaeiilaWIaeq4Wdm3aaWbaaSqabeaacqaIYaGmaaGccqGG8baFcqWG5bqEcqGGPaqkcqGHDisTaeaacqWGWbaCcqGGOaakcqaH8oqBcqGGSaalcqqHuoarcqaH8oqBcqGGSaalcqaHdpWCdaahaaWcbeqaaiabikdaYaaakiabcMcaPmaarahabaWaaSaaaeaacqaIXaqmaeaadaGcaaqaaiabikdaYiabec8aWbWcbeaakiabeo8aZbaaaSqaaiabdMgaPjabg2da9iabigdaXaqaaiabd6gaUnaaBaaameaacqaIXaqmaeqaaaqdcqGHpis1aOGagiyzauMaeiiEaGNaeiiCaa3aaeWaaeaacqGHsisldaWcaaqaaiabigdaXaqaaiabikdaYiabeo8aZnaaCaaaleqabaGaeGOmaidaaaaakmaabmaabaGaemyEaK3aaSbaaSqaaiabdMgaPbqabaGccqGHsislcqaH8oqBaiaawIcacaGLPaaadaahaaWcbeqaaiabikdaYaaaaOGaayjkaiaawMcaamaarahabaWaaSaaaeaacqaIXaqmaeaadaGcaaqaaiabikdaYiabec8aWbWcbeaakiabeo8aZbaacyGGLbqzcqGG4baEcqGGWbaCaSqaaiabdQgaQjabg2da9iabigdaXaqaaiabd6gaUnaaBaaameaacqaIYaGmaeqaaaqdcqGHpis1aOWaaeWaaeaacqGHsisldaWcaaqaaiabigdaXaqaaiabikdaYiabeo8aZnaaCaaaleqabaGaeGOmaidaaaaakmaabmaabaGaemyEaK3aaSbaaSqaaiabdQgaQbqabaGccqGHsislcqGGOaakcqaH8oqBcqGHRaWkcqqHuoarcqaH8oqBcqGGPaqkaiaawIcacaGLPaaadaahaaWcbeqaaiabikdaYaaaaOGaayjkaiaawMcaaaaacaWLjaGaaCzcamaabmaabaGaeGOmaiJaeG4mamdacaGLOaGaayzkaaaaaa@97EB@

Combining the joint likelihood into a common exponential gives:

p(μ,Δμ,σ2|y)∝p(μ,Δμ,σ2)σ−(n1+n2)exp⁡(−12σ2[∑i=1n1(yi−μ)2+∑j=1n2(yj−(μ+Δμ))2])     (24)
 MathType@MTEF@5@5@+=feaafiart1ev1aaatCvAUfKttLearuWrP9MDH5MBPbIqV92AaeXatLxBI9gBaebbnrfifHhDYfgasaacH8akY=wiFfYdH8Gipec8Eeeu0xXdbba9frFj0=OqFfea0dXdd9vqai=hGuQ8kuc9pgc9s8qqaq=dirpe0xb9q8qiLsFr0=vr0=vr0dc8meaabaqaciaacaGaaeqabaqabeGadaaakeaacqWGWbaCcqGGOaakcqaH8oqBcqGGSaalcqqHuoarcqaH8oqBcqGGSaalcqaHdpWCdaahaaWcbeqaaiabikdaYaaakiabcYha8jabdMha5jabcMcaPiabg2Hi1kabdchaWjabcIcaOiabeY7aTjabcYcaSiabfs5aejabeY7aTjabcYcaSiabeo8aZnaaCaaaleqabaGaeGOmaidaaOGaeiykaKIaeq4Wdm3aaWbaaSqabeaacqGHsislcqGGOaakcqWGUbGBdaWgaaadbaGaeGymaedabeaaliabgUcaRiabd6gaUnaaBaaameaacqaIYaGmaeqaaSGaeiykaKcaaOGagiyzauMaeiiEaGNaeiiCaa3aaeWaaeaacqGHsisldaWcaaqaaiabigdaXaqaaiabikdaYiabeo8aZnaaCaaaleqabaGaeGOmaidaaaaakmaadmaabaWaaabCaeaadaqadaqaaiabdMha5naaBaaaleaacqWGPbqAaeqaaOGaeyOeI0IaeqiVd0gacaGLOaGaayzkaaWaaWbaaSqabeaacqaIYaGmaaGccqGHRaWkdaaeWbqaamaabmaabaGaemyEaK3aaSbaaSqaaiabdQgaQbqabaGccqGHsislcqGGOaakcqaH8oqBcqGHRaWkcqqHuoarcqaH8oqBcqGGPaqkaiaawIcacaGLPaaadaahaaWcbeqaaiabikdaYaaaaeaacqWGQbGAcqGH9aqpcqaIXaqmaeaacqWGUbGBdaWgaaadbaGaeGOmaidabeaaa0GaeyyeIuoaaSqaaiabdMgaPjabg2da9iabigdaXaqaaiabd6gaUnaaBaaameaacqaIXaqmaeqaaaqdcqGHris5aaGccaGLBbGaayzxaaaacaGLOaGaayzkaaGaaCzcaiaaxMaadaqadaqaaiabikdaYiabisda0aGaayjkaiaawMcaaaaa@8E98@

Next we can define a variable that that only contains terms for the expression levels:

Cμ=∑i=1n1(yi−μ)2+∑j=1n2(yj−(μ+Δμ))2     (25)
 MathType@MTEF@5@5@+=feaafiart1ev1aaatCvAUfKttLearuWrP9MDH5MBPbIqV92AaeXatLxBI9gBaebbnrfifHhDYfgasaacH8akY=wiFfYdH8Gipec8Eeeu0xXdbba9frFj0=OqFfea0dXdd9vqai=hGuQ8kuc9pgc9s8qqaq=dirpe0xb9q8qiLsFr0=vr0=vr0dc8meaabaqaciaacaGaaeqabaqabeGadaaakeaacqWGdbWqdaWgaaWcbaGaeqiVd0gabeaakiabg2da9maaqahabaWaaeWaaeaacqWG5bqEdaWgaaWcbaGaemyAaKgabeaakiabgkHiTiabeY7aTbGaayjkaiaawMcaamaaCaaaleqabaGaeGOmaidaaOGaey4kaSYaaabCaeaadaqadaqaaiabdMha5naaBaaaleaacqWGQbGAaeqaaOGaeyOeI0IaeiikaGIaeqiVd0Maey4kaSIaeuiLdqKaeqiVd0MaeiykaKcacaGLOaGaayzkaaWaaWbaaSqabeaacqaIYaGmaaaabaGaemOAaOMaeyypa0JaeGymaedabaGaemOBa42aaSbaaWqaaiabikdaYaqabaaaniabggHiLdaaleaacqWGPbqAcqGH9aqpcqaIXaqmaeaacqWGUbGBdaWgaaadbaGaeGymaedabeaaa0GaeyyeIuoakiaaxMaacaWLjaWaaeWaaeaacqaIYaGmcqaI1aqnaiaawIcacaGLPaaaaaa@5CDD@

p(μ,Δμ,σ2|y)∝p(μ,Δμ,σ2)σ−(n1+n2)exp⁡(−Cμ2σ2)     (26)
 MathType@MTEF@5@5@+=feaafiart1ev1aaatCvAUfKttLearuWrP9MDH5MBPbIqV92AaeXatLxBI9gBaebbnrfifHhDYfgasaacH8akY=wiFfYdH8Gipec8Eeeu0xXdbba9frFj0=OqFfea0dXdd9vqai=hGuQ8kuc9pgc9s8qqaq=dirpe0xb9q8qiLsFr0=vr0=vr0dc8meaabaqaciaacaGaaeqabaqabeGadaaakeaacqWGWbaCcqGGOaakcqaH8oqBcqGGSaalcqqHuoarcqaH8oqBcqGGSaalcqaHdpWCdaahaaWcbeqaaiabikdaYaaakiabcYha8jabdMha5jabcMcaPiabg2Hi1kabdchaWjabcIcaOiabeY7aTjabcYcaSiabfs5aejabeY7aTjabcYcaSiabeo8aZnaaCaaaleqabaGaeGOmaidaaOGaeiykaKIaeq4Wdm3aaWbaaSqabeaacqGHsislcqGGOaakcqWGUbGBdaWgaaadbaGaeGymaedabeaaliabgUcaRiabd6gaUnaaBaaameaacqaIYaGmaeqaaSGaeiykaKcaaOGagiyzauMaeiiEaGNaeiiCaa3aaeWaaeaacqGHsisldaWcaaqaaiabdoeadnaaBaaaleaacqaH8oqBaeqaaaGcbaGaeGOmaiJaeq4Wdm3aaWbaaSqabeaacqaIYaGmaaaaaaGccaGLOaGaayzkaaGaaCzcaiaaxMaadaqadaqaaiabikdaYiabiAda2aGaayjkaiaawMcaaaaa@673C@

Expanding the square leads to the following:

Cμ=∑i=1n1(yi2−2yiμ+μ2)+∑j=1n2(yj2−2yjμ−2yjΔμ+μ2+Δμ2+2μΔμ)     (27)
 MathType@MTEF@5@5@+=feaafiart1ev1aaatCvAUfKttLearuWrP9MDH5MBPbIqV92AaeXatLxBI9gBaebbnrfifHhDYfgasaacH8akY=wiFfYdH8Gipec8Eeeu0xXdbba9frFj0=OqFfea0dXdd9vqai=hGuQ8kuc9pgc9s8qqaq=dirpe0xb9q8qiLsFr0=vr0=vr0dc8meaabaqaciaacaGaaeqabaqabeGadaaakeaacqWGdbWqdaWgaaWcbaGaeqiVd0gabeaakiabg2da9maaqahabaWaaeWaaeaacqWG5bqEdaqhaaWcbaGaemyAaKgabaGaeGOmaidaaOGaeyOeI0IaeGOmaiJaemyEaK3aaSbaaSqaaiabdMgaPbqabaGccqaH8oqBcqGHRaWkcqaH8oqBdaahaaWcbeqaaiabikdaYaaaaOGaayjkaiaawMcaaaWcbaGaemyAaKMaeyypa0JaeGymaedabaGaemOBa42aaSbaaWqaaiabigdaXaqabaaaniabggHiLdGccqGHRaWkdaaeWbqaamaabmaabaGaemyEaK3aa0baaSqaaiabdQgaQbqaaiabikdaYaaakiabgkHiTiabikdaYiabdMha5naaBaaaleaacqWGQbGAaeqaaOGaeqiVd0MaeyOeI0IaeGOmaiJaemyEaK3aaSbaaSqaaiabdQgaQbqabaGccqqHuoarcqaH8oqBcqGHRaWkcqaH8oqBdaahaaWcbeqaaiabikdaYaaakiabgUcaRiabfs5aejabeY7aTnaaCaaaleqabaGaeGOmaidaaOGaey4kaSIaeGOmaiJaeqiVd0MaeuiLdqKaeqiVd0gacaGLOaGaayzkaaaaleaacqWGQbGAcqGH9aqpcqaIXaqmaeaacqWGUbGBdaWgaaadbaGaeGOmaidabeaaa0GaeyyeIuoakiaaxMaacaWLjaWaaeWaaeaacqaIYaGmcqaI3aWnaiaawIcacaGLPaaaaaa@7A3A@

y¯1=1n1∑i=1n1yi     (28)
 MathType@MTEF@5@5@+=feaafiart1ev1aaatCvAUfKttLearuWrP9MDH5MBPbIqV92AaeXatLxBI9gBaebbnrfifHhDYfgasaacH8akY=wiFfYdH8Gipec8Eeeu0xXdbba9frFj0=OqFfea0dXdd9vqai=hGuQ8kuc9pgc9s8qqaq=dirpe0xb9q8qiLsFr0=vr0=vr0dc8meaabaqaciaacaGaaeqabaqabeGadaaakeaacuWG5bqEgaqeamaaBaaaleaacqaIXaqmaeqaaOGaeyypa0ZaaSaaaeaacqaIXaqmaeaacqWGUbGBdaWgaaWcbaGaeGymaedabeaaaaGcdaaeWbqaaiabdMha5naaBaaaleaacqWGPbqAaeqaaaqaaiabdMgaPjabg2da9iabigdaXaqaaiabd6gaUnaaBaaameaacqaIXaqmaeqaaaqdcqGHris5aOGaaCzcaiaaxMaadaqadaqaaiabikdaYiabiIda4aGaayjkaiaawMcaaaaa@43C9@

y¯2=1n2∑j=1n2yj     (29)
 MathType@MTEF@5@5@+=feaafiart1ev1aaatCvAUfKttLearuWrP9MDH5MBPbIqV92AaeXatLxBI9gBaebbnrfifHhDYfgasaacH8akY=wiFfYdH8Gipec8Eeeu0xXdbba9frFj0=OqFfea0dXdd9vqai=hGuQ8kuc9pgc9s8qqaq=dirpe0xb9q8qiLsFr0=vr0=vr0dc8meaabaqaciaacaGaaeqabaqabeGadaaakeaacuWG5bqEgaqeamaaBaaaleaacqaIYaGmaeqaaOGaeyypa0ZaaSaaaeaacqaIXaqmaeaacqWGUbGBdaWgaaWcbaGaeGOmaidabeaaaaGcdaaeWbqaaiabdMha5naaBaaaleaacqWGQbGAaeqaaaqaaiabdQgaQjabg2da9iabigdaXaqaaiabd6gaUnaaBaaameaacqaIYaGmaeqaaaqdcqGHris5aOGaaCzcaiaaxMaadaqadaqaaiabikdaYiabiMda5aGaayjkaiaawMcaaaaa@43D5@

Cμ=(n1+n2)μ2−2(n1y¯1+n2y¯2−n2Δμ)μ+∑i=1n1yj2+∑j=1n2yj2+n2Δμ2−2n2y¯2Δμ     (30)
 MathType@MTEF@5@5@+=feaafiart1ev1aaatCvAUfKttLearuWrP9MDH5MBPbIqV92AaeXatLxBI9gBaebbnrfifHhDYfgasaacH8akY=wiFfYdH8Gipec8Eeeu0xXdbba9frFj0=OqFfea0dXdd9vqai=hGuQ8kuc9pgc9s8qqaq=dirpe0xb9q8qiLsFr0=vr0=vr0dc8meaabaqaciaacaGaaeqabaqabeGadaaakeaacqWGdbWqdaWgaaWcbaGaeqiVd0gabeaakiabg2da9iabcIcaOiabd6gaUnaaBaaaleaacqaIXaqmaeqaaOGaey4kaSIaemOBa42aaSbaaSqaaiabikdaYaqabaGccqGGPaqkcqaH8oqBdaahaaWcbeqaaiabikdaYaaakiabgkHiTiabikdaYiabcIcaOiabd6gaUnaaBaaaleaacqaIXaqmaeqaaOGafmyEaKNbaebadaWgaaWcbaGaeGymaedabeaakiabgUcaRiabd6gaUnaaBaaaleaacqaIYaGmaeqaaOGafmyEaKNbaebadaWgaaWcbaGaeGOmaidabeaakiabgkHiTiabd6gaUnaaBaaaleaacqaIYaGmaeqaaOGaeuiLdqKaeqiVd0MaeiykaKIaeqiVd0Maey4kaSYaaabCaeaacqWG5bqEdaqhaaWcbaGaemOAaOgabaGaeGOmaidaaOGaey4kaSYaaabCaeaacqWG5bqEdaqhaaWcbaGaemOAaOgabaGaeGOmaidaaaqaaiabdQgaQjabg2da9iabigdaXaqaaiabd6gaUnaaBaaameaacqaIYaGmaeqaaaqdcqGHris5aOGaey4kaSIaemOBa42aaSbaaSqaaiabikdaYaqabaGccqqHuoarcqaH8oqBdaahaaWcbeqaaiabikdaYaaakiabgkHiTiabikdaYiabd6gaUnaaBaaaleaacqaIYaGmaeqaaOGafmyEaKNbaebadaWgaaWcbaGaeGOmaidabeaakiabfs5aejabeY7aTbWcbaGaemyAaKMaeyypa0JaeGymaedabaGaemOBa42aaSbaaWqaaiabigdaXaqabaaaniabggHiLdGccaWLjaGaaCzcamaabmaabaGaeG4mamJaeGimaadacaGLOaGaayzkaaaaaa@831C@

Next, we look to complete the square by adding and subtracting the same value *k*:

k=n1y¯1+n2y¯2−n2Δμn1+n2     (31)
 MathType@MTEF@5@5@+=feaafiart1ev1aaatCvAUfKttLearuWrP9MDH5MBPbIqV92AaeXatLxBI9gBaebbnrfifHhDYfgasaacH8akY=wiFfYdH8Gipec8Eeeu0xXdbba9frFj0=OqFfea0dXdd9vqai=hGuQ8kuc9pgc9s8qqaq=dirpe0xb9q8qiLsFr0=vr0=vr0dc8meaabaqaciaacaGaaeqabaqabeGadaaakeaacqWGRbWAcqGH9aqpdaWcaaqaaiabd6gaUnaaBaaaleaacqaIXaqmaeqaaOGafmyEaKNbaebadaWgaaWcbaGaeGymaedabeaakiabgUcaRiabd6gaUnaaBaaaleaacqaIYaGmaeqaaOGafmyEaKNbaebadaWgaaWcbaGaeGOmaidabeaakiabgkHiTiabd6gaUnaaBaaaleaacqaIYaGmaeqaaOGaeuiLdqKaeqiVd0gabaGaemOBa42aaSbaaSqaaiabigdaXaqabaGccqGHRaWkcqWGUbGBdaWgaaWcbaGaeGOmaidabeaaaaGccaWLjaGaaCzcamaabmaabaGaeG4mamJaeGymaedacaGLOaGaayzkaaaaaa@4BD0@

Cμ=(n1+n2)(μ2−2kμ+k2−k2)+∑i=1n1yi2+∑j=1n2yj2+n2Δμ2−2n2y¯2Δμ     (32)
 MathType@MTEF@5@5@+=feaafiart1ev1aaatCvAUfKttLearuWrP9MDH5MBPbIqV92AaeXatLxBI9gBaebbnrfifHhDYfgasaacH8akY=wiFfYdH8Gipec8Eeeu0xXdbba9frFj0=OqFfea0dXdd9vqai=hGuQ8kuc9pgc9s8qqaq=dirpe0xb9q8qiLsFr0=vr0=vr0dc8meaabaqaciaacaGaaeqabaqabeGadaaakeaacqWGdbWqdaWgaaWcbaGaeqiVd0gabeaakiabg2da9iabcIcaOiabd6gaUnaaBaaaleaacqaIXaqmaeqaaOGaey4kaSIaemOBa42aaSbaaSqaaiabikdaYaqabaGccqGGPaqkdaqadaqaaiabeY7aTnaaCaaaleqabaGaeGOmaidaaOGaeyOeI0IaeGOmaiJaem4AaSMaeqiVd0Maey4kaSIaem4AaS2aaWbaaSqabeaacqaIYaGmaaGccqGHsislcqWGRbWAdaahaaWcbeqaaiabikdaYaaaaOGaayjkaiaawMcaaiabgUcaRmaaqahabaGaemyEaK3aa0baaSqaaiabdMgaPbqaaiabikdaYaaakiabgUcaRmaaqahabaGaemyEaK3aa0baaSqaaiabdQgaQbqaaiabikdaYaaakiabgUcaRiabd6gaUnaaBaaaleaacqaIYaGmaeqaaOGaeuiLdqKaeqiVd02aaWbaaSqabeaacqaIYaGmaaGccqGHsislcqaIYaGmcqWGUbGBdaWgaaWcbaGaeGOmaidabeaakiqbdMha5zaaraWaaSbaaSqaaiabikdaYaqabaGccqqHuoarcqaH8oqBaSqaaiabdQgaQjabg2da9iabigdaXaqaaiabd6gaUnaaBaaameaacqaIYaGmaeqaaaqdcqGHris5aaWcbaGaemyAaKMaeyypa0JaeGymaedabaGaemOBa42aaSbaaWqaaiabigdaXaqabaaaniabggHiLdGccaWLjaGaaCzcamaabmaabaGaeG4mamJaeGOmaidacaGLOaGaayzkaaaaaa@793A@

Now collapsing back to a single squared term in the exponent and defining a new variable, *C*_σ_, we have

*C*_μ _= (*n*_1 _+ *n*_2_)(μ - *k*)^2 ^+ *C*_σ _    (33)

where

Cσ=∑i=1n1yi2+∑j=1n2yj2+n2Δμ2−2n2y¯2Δμ−(n1+n2)k2     (34)
 MathType@MTEF@5@5@+=feaafiart1ev1aaatCvAUfKttLearuWrP9MDH5MBPbIqV92AaeXatLxBI9gBaebbnrfifHhDYfgasaacH8akY=wiFfYdH8Gipec8Eeeu0xXdbba9frFj0=OqFfea0dXdd9vqai=hGuQ8kuc9pgc9s8qqaq=dirpe0xb9q8qiLsFr0=vr0=vr0dc8meaabaqaciaacaGaaeqabaqabeGadaaakeaacqWGdbWqdaWgaaWcbaGaeq4Wdmhabeaakiabg2da9maaqahabaGaemyEaK3aa0baaSqaaiabdMgaPbqaaiabikdaYaaakiabgUcaRmaaqahabaGaemyEaK3aa0baaSqaaiabdQgaQbqaaiabikdaYaaakiabgUcaRiabd6gaUnaaBaaaleaacqaIYaGmaeqaaOGaeuiLdqKaeqiVd02aaWbaaSqabeaacqaIYaGmaaGccqGHsislcqaIYaGmcqWGUbGBdaWgaaWcbaGaeGOmaidabeaakiqbdMha5zaaraWaaSbaaSqaaiabikdaYaqabaGccqqHuoarcqaH8oqBcqGHsislcqGGOaakcqWGUbGBdaWgaaWcbaGaeGymaedabeaakiabgUcaRiabd6gaUnaaBaaaleaacqaIYaGmaeqaaOGaeiykaKIaem4AaS2aaWbaaSqabeaacqaIYaGmaaaabaGaemOAaOMaeyypa0JaeGymaedabaGaemOBa42aaSbaaWqaaiabikdaYaqabaaaniabggHiLdaaleaacqWGPbqAcqGH9aqpcqaIXaqmaeaacqWGUbGBdaWgaaadbaGaeGymaedabeaaa0GaeyyeIuoakiaaxMaacaWLjaWaaeWaaeaacqaIZaWmcqaI0aanaiaawIcacaGLPaaaaaa@6B8E@

The posterior density can then be written as

p(μ,Δμ,σ2|y)∝p(μ,Δμ,σ2)σ−(n1+n2)exp⁡(−(n1+n2)2σ2(μ−k)2)exp⁡(−Cσ2σ2)     (35)
 MathType@MTEF@5@5@+=feaafiart1ev1aaatCvAUfKttLearuWrP9MDH5MBPbIqV92AaeXatLxBI9gBaebbnrfifHhDYfgasaacH8akY=wiFfYdH8Gipec8Eeeu0xXdbba9frFj0=OqFfea0dXdd9vqai=hGuQ8kuc9pgc9s8qqaq=dirpe0xb9q8qiLsFr0=vr0=vr0dc8meaabaqaciaacaGaaeqabaqabeGadaaakeaacqWGWbaCcqGGOaakcqaH8oqBcqGGSaalcqqHuoarcqaH8oqBcqGGSaalcqaHdpWCdaahaaWcbeqaaiabikdaYaaakiabcYha8jabdMha5jabcMcaPiabg2Hi1kabdchaWjabcIcaOiabeY7aTjabcYcaSiabfs5aejabeY7aTjabcYcaSiabeo8aZnaaCaaaleqabaGaeGOmaidaaOGaeiykaKIaeq4Wdm3aaWbaaSqabeaacqGHsislcqGGOaakcqWGUbGBdaWgaaadbaGaeGymaedabeaaliabgUcaRiabd6gaUnaaBaaameaacqaIYaGmaeqaaSGaeiykaKcaaOGagiyzauMaeiiEaGNaeiiCaa3aaeWaaeaacqGHsisldaWcaaqaaiabcIcaOiabd6gaUnaaBaaaleaacqaIXaqmaeqaaOGaey4kaSIaemOBa42aaSbaaSqaaiabikdaYaqabaGccqGGPaqkaeaacqaIYaGmcqaHdpWCdaahaaWcbeqaaiabikdaYaaaaaGccqGGOaakcqaH8oqBcqGHsislcqWGRbWAcqGGPaqkdaahaaWcbeqaaiabikdaYaaaaOGaayjkaiaawMcaaiGbcwgaLjabcIha4jabcchaWnaabmaabaGaeyOeI0YaaSaaaeaacqWGdbWqdaWgaaWcbaGaeq4WdmhabeaaaOqaaiabikdaYiabeo8aZnaaCaaaleqabaGaeGOmaidaaaaaaOGaayjkaiaawMcaaiaaxMaacaWLjaWaaeWaaeaacqaIZaWmcqaI1aqnaiaawIcacaGLPaaaaaa@806A@

We can now look to integrate out the true expression level, μ:

p(Δμ,σ2|y)∝∫−∞+∞p(μ,Δμ,σ2|y)dμ     (36)
MathType@MTEF@5@5@+=feaafiart1ev1aaatCvAUfKttLearuWrP9MDH5MBPbIqV92AaeXatLxBI9gBaebbnrfifHhDYfgasaacH8akY=wiFfYdH8Gipec8Eeeu0xXdbba9frFj0=OqFfea0dXdd9vqai=hGuQ8kuc9pgc9s8qqaq=dirpe0xb9q8qiLsFr0=vr0=vr0dc8meaabaqaciaacaGaaeqabaqabeGadaaakeaacqWGWbaCcqGGOaakcqqHuoarcqaH8oqBcqGGSaalcqaHdpWCdaahaaWcbeqaaiabikdaYaaakiabcYha8jabdMha5jabcMcaPiabg2Hi1oaapehabaGaemiCaaNaeiikaGIaeqiVd0MaeiilaWIaeuiLdqKaeqiVd0MaeiilaWIaeq4Wdm3aaWbaaSqabeaacqaIYaGmaaGccqGG8baFcqWG5bqEcqGGPaqkcqWGKbazcqaH8oqBaSqaaiabgkHiTiabg6HiLcqaaiabgUcaRiabg6HiLcqdcqGHRiI8aOGaaCzcaiaaxMaadaqadaqaaiabiodaZiabiAda2aGaayjkaiaawMcaaaaa@59A4@

We have flat priors on μ and Δμ, allowing us to complete the integration over all μ.

*p*(μ,Δμ,σ^2^) ∝ *p*(σ^2^)     (37)

p(Δμ,σ2|y)∝p(σ2)σ−(n1+n2)exp⁡(−Cσ2σ2)∫−∞+∞exp⁡(−(n1+n2)2σ2(μ−k)2)dμ     (38)
MathType@MTEF@5@5@+=feaafiart1ev1aaatCvAUfKttLearuWrP9MDH5MBPbIqV92AaeXatLxBI9gBaebbnrfifHhDYfgasaacH8akY=wiFfYdH8Gipec8Eeeu0xXdbba9frFj0=OqFfea0dXdd9vqai=hGuQ8kuc9pgc9s8qqaq=dirpe0xb9q8qiLsFr0=vr0=vr0dc8meaabaqaciaacaGaaeqabaqabeGadaaakeaacqWGWbaCcqGGOaakcqqHuoarcqaH8oqBcqGGSaalcqaHdpWCdaahaaWcbeqaaiabikdaYaaakiabcYha8jabdMha5jabcMcaPiabg2Hi1kabdchaWjabcIcaOiabeo8aZnaaCaaaleqabaGaeGOmaidaaOGaeiykaKIaeq4Wdm3aaWbaaSqabeaacqGHsislcqGGOaakcqWGUbGBdaWgaaadbaGaeGymaedabeaaliabgUcaRiabd6gaUnaaBaaameaacqaIYaGmaeqaaSGaeiykaKcaaOGagiyzauMaeiiEaGNaeiiCaa3aaeWaaeaacqGHsisldaWcaaqaaiabdoeadnaaBaaaleaacqaHdpWCaeqaaaGcbaGaeGOmaiJaeq4Wdm3aaWbaaSqabeaacqaIYaGmaaaaaaGccaGLOaGaayzkaaWaa8qCaeaacyGGLbqzcqGG4baEcqGGWbaCaSqaaiabgkHiTiabg6HiLcqaaiabgUcaRiabg6HiLcqdcqGHRiI8aOWaaeWaaeaacqGHsisldaWcaaqaaiabcIcaOiabd6gaUnaaBaaaleaacqaIXaqmaeqaaOGaey4kaSIaemOBa42aaSbaaSqaaiabikdaYaqabaGccqGGPaqkaeaacqaIYaGmcqaHdpWCdaahaaWcbeqaaiabikdaYaaaaaGccqGGOaakcqaH8oqBcqGHsislcqWGRbWAcqGGPaqkdaahaaWcbeqaaiabikdaYaaaaOGaayjkaiaawMcaaiabdsgaKjabeY7aTjaaxMaacaWLjaWaaeWaaeaacqaIZaWmcqaI4aaoaiaawIcacaGLPaaaaaa@8173@

∫−∞+∞exp⁡(−(n1+n22σ2(μ−k)2)dμ∝σ     (39)
MathType@MTEF@5@5@+=feaafiart1ev1aaatCvAUfKttLearuWrP9MDH5MBPbIqV92AaeXatLxBI9gBaebbnrfifHhDYfgasaacH8akY=wiFfYdH8Gipec8Eeeu0xXdbba9frFj0=OqFfea0dXdd9vqai=hGuQ8kuc9pgc9s8qqaq=dirpe0xb9q8qiLsFr0=vr0=vr0dc8meaabaqaciaacaGaaeqabaqabeGadaaakeaadaWdXbqaaiGbcwgaLjabcIha4jabcchaWbWcbaGaeyOeI0IaeyOhIukabaGaey4kaSIaeyOhIukaniabgUIiYdGcdaqadaqaaiabgkHiTmaalaaabaGaeiikaGIaemOBa42aaSbaaSqaaiabigdaXaqabaGccqGHRaWkcqWGUbGBdaWgaaWcbaGaeGOmaidabeaaaOqaaiabikdaYiabeo8aZnaaCaaaleqabaGaeGOmaidaaaaakiabcIcaOiabeY7aTjabgkHiTiabdUgaRjabcMcaPmaaCaaaleqabaGaeGOmaidaaaGccaGLOaGaayzkaaGaemizaqMaeqiVd0MaeyyhIuRaeq4WdmNaaCzcaiaaxMaadaqadaqaaiabiodaZiabiMda5aGaayjkaiaawMcaaaaa@5722@

The posterior for the joint density of Δμ and σ^2 ^is then

p(Δμ,σ2|y)∝p(σ2)σ−(n1+n2−1)exp⁡(−Cσ2σ2)     (40)
 MathType@MTEF@5@5@+=feaafiart1ev1aaatCvAUfKttLearuWrP9MDH5MBPbIqV92AaeXatLxBI9gBaebbnrfifHhDYfgasaacH8akY=wiFfYdH8Gipec8Eeeu0xXdbba9frFj0=OqFfea0dXdd9vqai=hGuQ8kuc9pgc9s8qqaq=dirpe0xb9q8qiLsFr0=vr0=vr0dc8meaabaqaciaacaGaaeqabaqabeGadaaakeaacqWGWbaCcqGGOaakcqqHuoarcqaH8oqBcqGGSaalcqaHdpWCdaahaaWcbeqaaiabikdaYaaakiabcYha8jabdMha5jabcMcaPiabg2Hi1kabdchaWjabcIcaOiabeo8aZnaaCaaaleqabaGaeGOmaidaaOGaeiykaKIaeq4Wdm3aaWbaaSqabeaacqGHsislcqGGOaakcqWGUbGBdaWgaaadbaGaeGymaedabeaaliabgUcaRiabd6gaUnaaBaaameaacqaIYaGmaeqaaSGaeyOeI0IaeGymaeJaeiykaKcaaOGagiyzauMaeiiEaGNaeiiCaa3aaeWaaeaacqGHsisldaWcaaqaaiabdoeadnaaBaaaleaacqaHdpWCaeqaaaGcbaGaeGOmaiJaeq4Wdm3aaWbaaSqabeaacqaIYaGmaaaaaaGccaGLOaGaayzkaaGaaCzcaiaaxMaadaqadaqaaiabisda0iabicdaWaGaayjkaiaawMcaaaaa@5FF6@

We next look to integrate out σ^2 ^by defining the sufficient statistics associated with the variance:

(n1−1)s12=∑i=1n1(yi−y¯1)2=∑i=1n1yi2−2n1y¯12+n1y¯12=∑i=1n1yi2−n1y¯12     (41)
 MathType@MTEF@5@5@+=feaafiart1ev1aaatCvAUfKttLearuWrP9MDH5MBPbIqV92AaeXatLxBI9gBaebbnrfifHhDYfgasaacH8akY=wiFfYdH8Gipec8Eeeu0xXdbba9frFj0=OqFfea0dXdd9vqai=hGuQ8kuc9pgc9s8qqaq=dirpe0xb9q8qiLsFr0=vr0=vr0dc8meaabaqaciaacaGaaeqabaqabeGadaaakeaacqGGOaakcqWGUbGBdaWgaaWcbaGaeGymaedabeaakiabgkHiTiabigdaXiabcMcaPiabdohaZnaaDaaaleaacqaIXaqmaeaacqaIYaGmaaGccqGH9aqpdaaeWbqaaiabcIcaOiabdMha5naaBaaaleaacqWGPbqAaeqaaOGaeyOeI0IafmyEaKNbaebadaWgaaWcbaGaeGymaedabeaakiabcMcaPmaaCaaaleqabaGaeGOmaidaaaqaaiabdMgaPjabg2da9iabigdaXaqaaiabd6gaUnaaBaaameaacqaIXaqmaeqaaaqdcqGHris5aOGaeyypa0ZaaabCaeaacqWG5bqEdaqhaaWcbaGaemyAaKgabaGaeGOmaidaaaqaaiabdMgaPjabg2da9iabigdaXaqaaiabd6gaUnaaBaaameaacqaIXaqmaeqaaaqdcqGHris5aOGaeyOeI0IaeGOmaiJaemOBa42aaSbaaSqaaiabigdaXaqabaGccuWG5bqEgaqeamaaDaaaleaacqaIXaqmaeaacqaIYaGmaaGccqGHRaWkcqWGUbGBdaWgaaWcbaGaeGymaedabeaakiqbdMha5zaaraWaa0baaSqaaiabigdaXaqaaiabikdaYaaakiabg2da9maaqahabaGaemyEaK3aa0baaSqaaiabdMgaPbqaaiabikdaYaaakiabgkHiTiabd6gaUnaaBaaaleaacqaIXaqmaeqaaOGafmyEaKNbaebadaqhaaWcbaGaeGymaedabaGaeGOmaidaaaqaaiabdMgaPjabg2da9iabigdaXaqaaiabd6gaUnaaBaaameaacqaIXaqmaeqaaaqdcqGHris5aOGaaCzcaiaaxMaadaqadaqaaiabisda0iabigdaXaGaayjkaiaawMcaaaaa@7E0F@

(n2−1)s22=∑j=1n2yj2−n2y¯22     (42)
 MathType@MTEF@5@5@+=feaafiart1ev1aaatCvAUfKttLearuWrP9MDH5MBPbIqV92AaeXatLxBI9gBaebbnrfifHhDYfgasaacH8akY=wiFfYdH8Gipec8Eeeu0xXdbba9frFj0=OqFfea0dXdd9vqai=hGuQ8kuc9pgc9s8qqaq=dirpe0xb9q8qiLsFr0=vr0=vr0dc8meaabaqaciaacaGaaeqabaqabeGadaaakeaadaqadaqaaiabd6gaUnaaBaaaleaacqaIYaGmaeqaaOGaeyOeI0IaeGymaedacaGLOaGaayzkaaGaem4Cam3aa0baaSqaaiabikdaYaqaaiabikdaYaaakiabg2da9maaqahabaGaemyEaK3aa0baaSqaaiabdQgaQbqaaiabikdaYaaakiabgkHiTiabd6gaUnaaBaaaleaacqaIYaGmaeqaaOGafmyEaKNbaebadaqhaaWcbaGaeGOmaidabaGaeGOmaidaaaqaaiabdQgaQjabg2da9iabigdaXaqaaiabd6gaUnaaBaaameaacqaIYaGmaeqaaaqdcqGHris5aOGaaCzcaiaaxMaadaqadaqaaiabisda0iabikdaYaGaayjkaiaawMcaaaaa@4F1B@

Cσ=(n1−1)s12+(n2−1)s22+CΔμ     (43)
 MathType@MTEF@5@5@+=feaafiart1ev1aaatCvAUfKttLearuWrP9MDH5MBPbIqV92AaeXatLxBI9gBaebbnrfifHhDYfgasaacH8akY=wiFfYdH8Gipec8Eeeu0xXdbba9frFj0=OqFfea0dXdd9vqai=hGuQ8kuc9pgc9s8qqaq=dirpe0xb9q8qiLsFr0=vr0=vr0dc8meaabaqaciaacaGaaeqabaqabeGadaaakeaacqWGdbWqdaWgaaWcbaGaeq4Wdmhabeaakiabg2da9iabcIcaOiabd6gaUnaaBaaaleaacqaIXaqmaeqaaOGaeyOeI0IaeGymaeJaeiykaKIaem4Cam3aa0baaSqaaiabigdaXaqaaiabikdaYaaakiabgUcaRiabcIcaOiabd6gaUnaaBaaaleaacqaIYaGmaeqaaOGaeyOeI0IaeGymaeJaeiykaKIaem4Cam3aa0baaSqaaiabikdaYaqaaiabikdaYaaakiabgUcaRiabdoeadnaaBaaaleaacqqHuoarcqaH8oqBaeqaaOGaaCzcaiaaxMaadaqadaqaaiabisda0iabiodaZaGaayjkaiaawMcaaaaa@4EDE@

where

CΔμ=n1y¯12+n2y¯22+n2Δμ2−2n2y¯2Δμ−(n1+n2)k2     (44)
 MathType@MTEF@5@5@+=feaafiart1ev1aaatCvAUfKttLearuWrP9MDH5MBPbIqV92AaeXatLxBI9gBaebbnrfifHhDYfgasaacH8akY=wiFfYdH8Gipec8Eeeu0xXdbba9frFj0=OqFfea0dXdd9vqai=hGuQ8kuc9pgc9s8qqaq=dirpe0xb9q8qiLsFr0=vr0=vr0dc8meaabaqaciaacaGaaeqabaqabeGadaaakeaacqWGdbWqdaWgaaWcbaGaeuiLdqKaeqiVd0gabeaakiabg2da9iabd6gaUnaaBaaaleaacqaIXaqmaeqaaOGafmyEaKNbaebadaqhaaWcbaGaeGymaedabaGaeGOmaidaaOGaey4kaSIaemOBa42aaSbaaSqaaiabikdaYaqabaGccuWG5bqEgaqeamaaDaaaleaacqaIYaGmaeaacqaIYaGmaaGccqGHRaWkcqWGUbGBdaWgaaWcbaGaeGOmaidabeaakiabfs5aejabeY7aTnaaCaaaleqabaGaeGOmaidaaOGaeyOeI0IaeGOmaiJaemOBa42aaSbaaSqaaiabikdaYaqabaGccuWG5bqEgaqeamaaBaaaleaacqaIYaGmaeqaaOGaeuiLdqKaeqiVd0MaeyOeI0IaeiikaGIaemOBa42aaSbaaSqaaiabigdaXaqabaGccqGHRaWkcqWGUbGBdaWgaaWcbaGaeGOmaidabeaakiabcMcaPiabdUgaRnaaCaaaleqabaGaeGOmaidaaOGaaCzcaiaaxMaadaqadaqaaiabisda0iabisda0aGaayjkaiaawMcaaaaa@6138@

The last term in Eq. (43) *C*Δμ, now contains all references to the null difference in means, Δμ. The term can be expanded by multiplying out *k*^2 ^and then simplified:

CΔμ=n1y¯12+n2y¯22+n2Δμ2−2n2y¯2Δμ−1n1+n2(n12y¯12+2n1n2y¯1y¯2+n22y¯22−2n1n2y¯1Δμ+n22Δμ2−2n22y¯2Δμ)     45=n1n2n1+n2(Δμ−(y¯2−y¯1))2
 MathType@MTEF@5@5@+=feaafiart1ev1aaatCvAUfKttLearuWrP9MDH5MBPbIqV92AaeXatLxBI9gBaebbnrfifHhDYfgasaacH8akY=wiFfYdH8Gipec8Eeeu0xXdbba9frFj0=OqFfea0dXdd9vqai=hGuQ8kuc9pgc9s8qqaq=dirpe0xb9q8qiLsFr0=vr0=vr0dc8meaabaqaciaacaGaaeqabaqabeGadaaakqaaceqaaiabdoeadnaaBaaaleaacqGHuoarcqaH8oqBaeqaaOGaeyypa0JaemOBa42aaSbaaSqaaiabigdaXaqabaGccuWG5bqEgaqeamaaDaaaleaacqaIXaqmaeaacqaIYaGmaaGccqGHRaWkcqWGUbGBdaWgaaWcbaGaeGOmaidabeaakiqbdMha5zaaraWaa0baaSqaaiabikdaYaqaaiabikdaYaaakiabgUcaRiabd6gaUnaaBaaaleaacqaIYaGmaeqaaOGaeuiLdqKaeqiVd02aaWbaaSqabeaacqaIYaGmaaGccqGHsislcqaIYaGmcqWGUbGBdaWgaaWcbaGaeGOmaidabeaakiqbdMha5zaaraWaaSbaaSqaaiabikdaYaqabaGccqqHuoarcqaH8oqBcqGHsislaeaadaWcaaqaaiabigdaXaqaaiabd6gaUnaaBaaaleaacqaIXaqmaeqaaOGaey4kaSIaemOBa42aaSbaaSqaaiabikdaYaqabaaaaOWaaeWaaeaacqWGUbGBdaqhaaWcbaGaeGymaedabaGaeGOmaidaaOGafmyEaKNbaebadaqhaaWcbaGaeGymaedabaGaeGOmaidaaOGaey4kaSIaeGOmaiJaemOBa42aaSbaaSqaaiabigdaXaqabaGccqWGUbGBdaWgaaWcbaGaeGOmaidabeaakiqbdMha5zaaraWaaSbaaSqaaiabigdaXaqabaGccuWG5bqEgaqeamaaBaaaleaacqaIYaGmaeqaaOGaey4kaSIaemOBa42aa0baaSqaaiabikdaYaqaaiabikdaYaaakiqbdMha5zaaraWaa0baaSqaaiabikdaYaqaaiabikdaYaaakiabgkHiTiabikdaYiabd6gaUnaaBaaaleaacqaIXaqmaeqaaOGaemOBa42aaSbaaSqaaiabikdaYaqabaGccuWG5bqEgaqeamaaBaaaleaacqaIXaqmaeqaaOGaeuiLdqKaeqiVd0Maey4kaSIaemOBa42aa0baaSqaaiabikdaYaqaaiabikdaYaaakiabfs5aejabeY7aTnaaCaaaleqabaGaeGOmaidaaOGaeyOeI0IaeGOmaiJaemOBa42aa0baaSqaaiabikdaYaqaaiabikdaYaaakiqbdMha5zaaraWaaSbaaSqaaiabikdaYaqabaGccqqHuoardaWgaaWcbaGaeqiVd0gabeaaaOGaayjkaiaawMcaaiaaxMaacaWLjaGaeGinaqJaeGynaudabaGaeyypa0ZaaSaaaeaacqWGUbGBdaWgaaWcbaGaeGymaedabeaakiabd6gaUnaaBaaaleaacqaIYaGmaeqaaaGcbaGaemOBa42aaSbaaSqaaiabigdaXaqabaGccqGHRaWkcqWGUbGBdaWgaaWcbaGaeGOmaidabeaaaaGcdaqadaqaaiabfs5aejabeY7aTjabgkHiTiabcIcaOiqbdMha5zaaraWaaSbaaSqaaiabikdaYaqabaGccqGHsislcuWG5bqEgaqeamaaBaaaleaacqaIXaqmaeqaaOGaeiykaKcacaGLOaGaayzkaaWaaWbaaSqabeaacqaIYaGmaaaaaaa@B56F@

This can be inserted back into Eq. (43) to give

Cσ=(n1−1)s12+(n2−1)s22+n1n2n1+n2(Δμ−(y¯2−y¯2))2     (46)
 MathType@MTEF@5@5@+=feaafiart1ev1aaatCvAUfKttLearuWrP9MDH5MBPbIqV92AaeXatLxBI9gBaebbnrfifHhDYfgasaacH8akY=wiFfYdH8Gipec8Eeeu0xXdbba9frFj0=OqFfea0dXdd9vqai=hGuQ8kuc9pgc9s8qqaq=dirpe0xb9q8qiLsFr0=vr0=vr0dc8meaabaqaciaacaGaaeqabaqabeGadaaakeaacqWGdbWqdaWgaaWcbaGaeq4Wdmhabeaakiabg2da9iabcIcaOiabd6gaUnaaBaaaleaacqaIXaqmaeqaaOGaeyOeI0IaeGymaeJaeiykaKIaem4Cam3aa0baaSqaaiabigdaXaqaaiabikdaYaaakiabgUcaRiabcIcaOiabd6gaUnaaBaaaleaacqaIYaGmaeqaaOGaeyOeI0IaeGymaeJaeiykaKIaem4Cam3aa0baaSqaaiabikdaYaqaaiabikdaYaaakiabgUcaRmaalaaabaGaemOBa42aaSbaaSqaaiabigdaXaqabaGccqWGUbGBdaWgaaWcbaGaeGOmaidabeaaaOqaaiabd6gaUnaaBaaaleaacqaIXaqmaeqaaOGaey4kaSIaemOBa42aaSbaaSqaaiabikdaYaqabaaaaOWaaeWaaeaacqqHuoarcqaH8oqBcqGHsislcqGGOaakcuWG5bqEgaqeamaaBaaaleaacqaIYaGmaeqaaOGaeyOeI0IafmyEaKNbaebadaWgaaWcbaGaeGOmaidabeaakiabcMcaPaGaayjkaiaawMcaamaaCaaaleqabaGaeGOmaidaaOGaaCzcaiaaxMaadaqadaqaaiabisda0iabiAda2aGaayjkaiaawMcaaaaa@6475@

The prior for variance is said to follow a scaled inverse Chi-square distribution:

p(σ2)=I(σ2;ν0,σ02)∝σ−(ν0+2)exp⁡(−12σ2ν0σ02)     (47)
 MathType@MTEF@5@5@+=feaafiart1ev1aaatCvAUfKttLearuWrP9MDH5MBPbIqV92AaeXatLxBI9gBaebbnrfifHhDYfgasaacH8akY=wiFfYdH8Gipec8Eeeu0xXdbba9frFj0=OqFfea0dXdd9vqai=hGuQ8kuc9pgc9s8qqaq=dirpe0xb9q8qiLsFr0=vr0=vr0dc8meaabaqaciaacaGaaeqabaqabeGadaaakeaacqWGWbaCcqGGOaakcqaHdpWCdaahaaWcbeqaaiabikdaYaaakiabcMcaPiabg2da9iabdMeajjabcIcaOiabeo8aZnaaCaaaleqabaGaeGOmaidaaOGaei4oaSJaeqyVd42aaSbaaSqaaiabicdaWaqabaGccqGGSaalcqaHdpWCdaqhaaWcbaGaeGimaadabaGaeGOmaidaaOGaeiykaKIaeyyhIuRaeq4Wdm3aaWbaaSqabeaacqGHsislcqGGOaakcqaH9oGBdaWgaaadbaGaeGimaadabeaaliabgUcaRiabikdaYiabcMcaPaaakiGbcwgaLjabcIha4jabcchaWnaabmaabaGaeyOeI0YaaSaaaeaacqaIXaqmaeaacqaIYaGmcqaHdpWCdaahaaWcbeqaaiabikdaYaaaaaGccqaH9oGBdaWgaaWcbaGaeGimaadabeaakiabeo8aZnaaDaaaleaacqaIWaamaeaacqaIYaGmaaaakiaawIcacaGLPaaacaWLjaGaaCzcamaabmaabaGaeGinaqJaeG4naCdacaGLOaGaayzkaaaaaa@63CE@

The posterior density is then

p(Δμ,σ2|y)∝σ−(n1+n2+ν0+1)exp⁡(−12σ2[ν0σ02+(n1−1)s12+(n2−1)s22+n1n2n1+n2(Δμ−(y¯2−y¯1))2])     (48)
 MathType@MTEF@5@5@+=feaafiart1ev1aaatCvAUfKttLearuWrP9MDH5MBPbIqV92AaeXatLxBI9gBaebbnrfifHhDYfgasaacH8akY=wiFfYdH8Gipec8Eeeu0xXdbba9frFj0=OqFfea0dXdd9vqai=hGuQ8kuc9pgc9s8qqaq=dirpe0xb9q8qiLsFr0=vr0=vr0dc8meaabaqaciaacaGaaeqabaqabeGadaaakqaaceqaaiabdchaWjabcIcaOiabfs5aejabeY7aTjabcYcaSiabeo8aZnaaCaaaleqabaGaeGOmaidaaOGaeiiFaWNaemyEaKNaeiykaKIaeyyhIulabaGaeq4Wdm3aaWbaaSqabeaacqGHsislcqGGOaakcqWGUbGBdaWgaaadbaGaeGymaedabeaaliabgUcaRiabd6gaUnaaBaaameaacqaIYaGmaeqaaSGaey4kaSIaeqyVd42aaSbaaWqaaiabicdaWaqabaWccqGHRaWkcqaIXaqmcqGGPaqkaaGccyGGLbqzcqGG4baEcqGGWbaCdaqadaqaaiabgkHiTmaalaaabaGaeGymaedabaGaeGOmaiJaeq4Wdm3aaWbaaSqabeaacqaIYaGmaaaaaOWaamWaaeaacqaH9oGBdaWgaaWcbaGaeGimaadabeaakiabeo8aZnaaDaaaleaacqaIWaamaeaacqaIYaGmaaGccqGHRaWkcqGGOaakcqWGUbGBdaWgaaWcbaGaeGymaedabeaakiabgkHiTiabigdaXiabcMcaPiabdohaZnaaDaaaleaacqaIXaqmaeaacqaIYaGmaaGccqGHRaWkcqGGOaakcqWGUbGBdaWgaaWcbaGaeGOmaidabeaakiabgkHiTiabigdaXiabcMcaPiabdohaZnaaDaaaleaacqaIYaGmaeaacqaIYaGmaaGccqGHRaWkdaWcaaqaaiabd6gaUnaaBaaaleaacqaIXaqmaeqaaOGaemOBa42aaSbaaSqaaiabikdaYaqabaaakeaacqWGUbGBdaWgaaWcbaGaeGymaedabeaakiabgUcaRiabd6gaUnaaBaaaleaacqaIYaGmaeqaaaaakmaabmaabaGaeuiLdqKaeqiVd0MaeyOeI0IaeiikaGIafmyEaKNbaebadaWgaaWcbaGaeGOmaidabeaakiabgkHiTiqbdMha5zaaraWaaSbaaSqaaiabigdaXaqabaGccqGGPaqkaiaawIcacaGLPaaadaahaaWcbeqaaiabikdaYaaaaOGaay5waiaaw2faaaGaayjkaiaawMcaaiaaxMaacaWLjaWaaeWaaeaacqaI0aancqaI4aaoaiaawIcacaGLPaaaaaaa@942A@

But now we can integrate out σ^2^

p(Δμ|y)∝∫0∞p(Δμ,σ2|y)dσ2     (49)
MathType@MTEF@5@5@+=feaafiart1ev1aaatCvAUfKttLearuWrP9MDH5MBPbIqV92AaeXatLxBI9gBaebbnrfifHhDYfgasaacH8akY=wiFfYdH8Gipec8Eeeu0xXdbba9frFj0=OqFfea0dXdd9vqai=hGuQ8kuc9pgc9s8qqaq=dirpe0xb9q8qiLsFr0=vr0=vr0dc8meaabaqaciaacaGaaeqabaqabeGadaaakeaacqWGWbaCcqGGOaakcqqHuoarcqaH8oqBcqGG8baFcqWG5bqEcqGGPaqkcqGHDisTdaWdXbqaaiabdchaWjabcIcaOiabfs5aejabeY7aTjabcYcaSiabeo8aZnaaCaaaleqabaGaeGOmaidaaOGaeiiFaWNaemyEaKNaeiykaKIaemizaqMaeq4Wdm3aaWbaaSqabeaacqaIYaGmaaaabaGaeGimaadabaGaeyOhIukaniabgUIiYdGccaWLjaGaaCzcamaabmaabaGaeGinaqJaeGyoaKdacaGLOaGaayzkaaaaaa@5219@

With a change of variables

A=ν0σ02+(n1−1)s12+(n2−1)s22+n1n2n1+n2(Δμ−(y¯2−y¯1))2     (50)
 MathType@MTEF@5@5@+=feaafiart1ev1aaatCvAUfKttLearuWrP9MDH5MBPbIqV92AaeXatLxBI9gBaebbnrfifHhDYfgasaacH8akY=wiFfYdH8Gipec8Eeeu0xXdbba9frFj0=OqFfea0dXdd9vqai=hGuQ8kuc9pgc9s8qqaq=dirpe0xb9q8qiLsFr0=vr0=vr0dc8meaabaqaciaacaGaaeqabaqabeGadaaakeaacqWGbbqqcqGH9aqpcqaH9oGBdaWgaaWcbaGaeGimaadabeaakiabeo8aZnaaDaaaleaacqaIWaamaeaacqaIYaGmaaGccqGHRaWkcqGGOaakcqWGUbGBdaWgaaWcbaGaeGymaedabeaakiabgkHiTiabigdaXiabcMcaPiabdohaZnaaDaaaleaacqaIXaqmaeaacqaIYaGmaaGccqGHRaWkcqGGOaakcqWGUbGBdaWgaaWcbaGaeGOmaidabeaakiabgkHiTiabigdaXiabcMcaPiabdohaZnaaDaaaleaacqaIYaGmaeaacqaIYaGmaaGccqGHRaWkdaWcaaqaaiabd6gaUnaaBaaaleaacqaIXaqmaeqaaOGaemOBa42aaSbaaSqaaiabikdaYaqabaaakeaacqWGUbGBdaWgaaWcbaGaeGymaedabeaakiabgUcaRiabd6gaUnaaBaaaleaacqaIYaGmaeqaaaaakmaabmaabaGaeuiLdqKaeqiVd0MaeyOeI0IaeiikaGIafmyEaKNbaebadaWgaaWcbaGaeGOmaidabeaakiabgkHiTiqbdMha5zaaraWaaSbaaSqaaiabigdaXaqabaGccqGGPaqkaiaawIcacaGLPaaadaahaaWcbeqaaiabikdaYaaakiaaxMaacaWLjaWaaeWaaeaacqaI1aqncqaIWaamaiaawIcacaGLPaaaaaa@6A04@

z=A2σ2     (51)
 MathType@MTEF@5@5@+=feaafiart1ev1aaatCvAUfKttLearuWrP9MDH5MBPbIqV92AaeXatLxBI9gBaebbnrfifHhDYfgasaacH8akY=wiFfYdH8Gipec8Eeeu0xXdbba9frFj0=OqFfea0dXdd9vqai=hGuQ8kuc9pgc9s8qqaq=dirpe0xb9q8qiLsFr0=vr0=vr0dc8meaabaqaciaacaGaaeqabaqabeGadaaakeaacqWG6bGEcqGH9aqpdaWcaaqaaiabdgeabbqaaiabikdaYiabeo8aZnaaCaaaleqabaGaeGOmaidaaaaakiaaxMaacaWLjaWaaeWaaeaacqaI1aqncqaIXaqmaiaawIcacaGLPaaaaaa@38DD@

p(Δμ|y)∝∫0∞(A2z)−(n1+n2+ν0+1)/2exp⁡(−z)(A2z2)dz     (52)
MathType@MTEF@5@5@+=feaafiart1ev1aaatCvAUfKttLearuWrP9MDH5MBPbIqV92AaeXatLxBI9gBaebbnrfifHhDYfgasaacH8akY=wiFfYdH8Gipec8Eeeu0xXdbba9frFj0=OqFfea0dXdd9vqai=hGuQ8kuc9pgc9s8qqaq=dirpe0xb9q8qiLsFr0=vr0=vr0dc8meaabaqaciaacaGaaeqabaqabeGadaaakeaacqWGWbaCcqGGOaakcqqHuoarcqaH8oqBcqGG8baFcqWG5bqEcqGGPaqkcqGHDisTdaWdXbqaamaabmaabaWaaSaaaeaacqWGbbqqaeaacqaIYaGmcqWG6bGEaaaacaGLOaGaayzkaaaaleaacqaIWaamaeaacqGHEisPa0Gaey4kIipakmaaCaaaleqabaGaeyOeI0IaeiikaGIaemOBa42aaSbaaWqaaiabigdaXaqabaWccqGHRaWkcqWGUbGBdaWgaaadbaGaeGOmaidabeaaliabgUcaRiabe27aUnaaBaaameaacqaIWaamaeqaaSGaey4kaSIaeGymaeJaeiykaKIaei4la8IaeGOmaidaaOGagiyzauMaeiiEaGNaeiiCaaNaeiikaGIaeyOeI0IaemOEaONaeiykaKYaaeWaaeaadaWcaaqaaiabdgeabbqaaiabikdaYiabdQha6naaCaaaleqabaGaeGOmaidaaaaaaOGaayjkaiaawMcaaiabdsgaKjabdQha6jaaxMaacaWLjaWaaeWaaeaacqaI1aqncqaIYaGmaiaawIcacaGLPaaaaaa@6792@

p(Δμ|y)∝A−(n1+n2+ν0−1)/2∫0∞z(n1+n2+ν0−3)/2exp⁡(−z)dz     (53)
MathType@MTEF@5@5@+=feaafiart1ev1aaatCvAUfKttLearuWrP9MDH5MBPbIqV92AaeXatLxBI9gBaebbnrfifHhDYfgasaacH8akY=wiFfYdH8Gipec8Eeeu0xXdbba9frFj0=OqFfea0dXdd9vqai=hGuQ8kuc9pgc9s8qqaq=dirpe0xb9q8qiLsFr0=vr0=vr0dc8meaabaqaciaacaGaaeqabaqabeGadaaakeaacqWGWbaCcqGGOaakcqqHuoarcqaH8oqBcqGG8baFcqWG5bqEcqGGPaqkcqGHDisTcqWGbbqqdaahaaWcbeqaaiabgkHiTiabcIcaOiabd6gaUnaaBaaameaacqaIXaqmaeqaaSGaey4kaSIaemOBa42aaSbaaWqaaiabikdaYaqabaWccqGHRaWkcqaH9oGBdaWgaaadbaGaeGimaadabeaaliabgkHiTiabigdaXiabcMcaPiabc+caViabikdaYaaakmaapehabaGaemOEaO3aaWbaaSqabeaacqGGOaakcqWGUbGBdaWgaaadbaGaeGymaedabeaaliabgUcaRiabd6gaUnaaBaaameaacqaIYaGmaeqaaSGaey4kaSIaeqyVd42aaSbaaWqaaiabicdaWaqabaWccqGHsislcqaIZaWmcqGGPaqkcqGGVaWlcqaIYaGmaaaabaGaeGimaadabaGaeyOhIukaniabgUIiYdGccyGGLbqzcqGG4baEcqGGWbaCcqGGOaakcqGHsislcqWG6bGEcqGGPaqkcqWGKbazcqWG6bGEcaWLjaGaaCzcamaabmaabaGaeGynauJaeG4mamdacaGLOaGaayzkaaaaaa@6E23@

The posterior density then becomes the well known *t *distribution.

p(Δμ|y)∝[1+(Δμ−(y¯2−y¯1))2νn(1n1+1n2)σn2]−(νn+1)/2     (54)
 MathType@MTEF@5@5@+=feaafiart1ev1aaatCvAUfKttLearuWrP9MDH5MBPbIqV92AaeXatLxBI9gBaebbnrfifHhDYfgasaacH8akY=wiFfYdH8Gipec8Eeeu0xXdbba9frFj0=OqFfea0dXdd9vqai=hGuQ8kuc9pgc9s8qqaq=dirpe0xb9q8qiLsFr0=vr0=vr0dc8meaabaqaciaacaGaaeqabaqabeGadaaakeaacqWGWbaCcqGGOaakcqqHuoarcqaH8oqBcqGG8baFcqWG5bqEcqGGPaqkcqGHDisTdaWadaqaaiabigdaXiabgUcaRmaalaaabaWaaeWaaeaacqqHuoarcqaH8oqBcqGHsislcqGGOaakcuWG5bqEgaqeamaaBaaaleaacqaIYaGmaeqaaOGaeyOeI0IafmyEaKNbaebadaWgaaWcbaGaeGymaedabeaakiabcMcaPaGaayjkaiaawMcaamaaCaaaleqabaGaeGOmaidaaaGcbaGaeqyVd42aaSbaaSqaaiabd6gaUbqabaGcdaqadaqaamaalaaabaGaeGymaedabaGaemOBa42aaSbaaSqaaiabigdaXaqabaaaaOGaey4kaSYaaSaaaeaacqaIXaqmaeaacqWGUbGBdaWgaaWcbaGaeGOmaidabeaaaaaakiaawIcacaGLPaaacqaHdpWCdaqhaaWcbaGaemOBa4gabaGaeGOmaidaaaaaaOGaay5waiaaw2faamaaCaaaleqabaGaeyOeI0IaeiikaGIaeqyVd42aaSbaaWqaaiabd6gaUbqabaWccqGHRaWkcqaIXaqmcqGGPaqkcqGGVaWlcqaIYaGmaaGccaWLjaGaaCzcamaabmaabaGaeGynauJaeGinaqdacaGLOaGaayzkaaaaaa@69B7@

where

ν_*n *_= *n*_1 _+ *n*_2 _+ ν_0 _- 2     (55)

νnσn2=ν0σ02+(n1−1)s12+(n2−1)s22     (56)
 MathType@MTEF@5@5@+=feaafiart1ev1aaatCvAUfKttLearuWrP9MDH5MBPbIqV92AaeXatLxBI9gBaebbnrfifHhDYfgasaacH8akY=wiFfYdH8Gipec8Eeeu0xXdbba9frFj0=OqFfea0dXdd9vqai=hGuQ8kuc9pgc9s8qqaq=dirpe0xb9q8qiLsFr0=vr0=vr0dc8meaabaqaciaacaGaaeqabaqabeGadaaakeaacqaH9oGBdaWgaaWcbaGaemOBa4gabeaakiabeo8aZnaaDaaaleaacqWGUbGBaeaacqaIYaGmaaGccqGH9aqpcqaH9oGBdaWgaaWcbaGaeGimaadabeaakiabeo8aZnaaDaaaleaacqaIWaamaeaacqaIYaGmaaGccqGHRaWkcqGGOaakcqWGUbGBdaWgaaWcbaGaeGymaedabeaakiabgkHiTiabigdaXiabcMcaPiabdohaZnaaDaaaleaacqaIXaqmaeaacqaIYaGmaaGccqGHRaWkcqGGOaakcqWGUbGBdaWgaaWcbaGaeGOmaidabeaakiabgkHiTiabigdaXiabcMcaPiabdohaZnaaDaaaleaacqaIYaGmaeaacqaIYaGmaaGccaWLjaGaaCzcamaabmaabaGaeGynauJaeGOnaydacaGLOaGaayzkaaaaaa@55D7@

Δμ−Δy¯σn1n1+1n2|y~tνn     (57)
 MathType@MTEF@5@5@+=feaafiart1ev1aaatCvAUfKttLearuWrP9MDH5MBPbIqV92AaeXatLxBI9gBaebbnrfifHhDYfgasaacH8akY=wiFfYdH8Gipec8Eeeu0xXdbba9frFj0=OqFfea0dXdd9vqai=hGuQ8kuc9pgc9s8qqaq=dirpe0xb9q8qiLsFr0=vr0=vr0dc8meaabaqaciaacaGaaeqabaqabeGadaaakeaadaabcaqaamaalaaabaGaeuiLdqKaeqiVd0MaeyOeI0IaeuiLdqKafmyEaKNbaebaaeaacqaHdpWCdaWgaaWcbaGaemOBa4gabeaakmaakaaabaWaaSaaaeaacqaIXaqmaeaacqWGUbGBdaWgaaWcbaGaeGymaedabeaaaaGccqGHRaWkdaWcaaqaaiabigdaXaqaaiabd6gaUnaaBaaaleaacqaIYaGmaeqaaaaaaeqaaaaaaOGaayjcSdGaemyEaKNaeiOFa4NaemiDaq3aaSbaaSqaaiabe27aUnaaBaaameaacqWGUbGBaeqaaaWcbeaakiaaxMaacaWLjaWaaeWaaeaacqaI1aqncqaI3aWnaiaawIcacaGLPaaaaaa@4D78@
